# Correction: SIRT1 Activity Is Linked to Its Brain Region-Specific Phosphorylation and Is Impaired in Huntington's Disease Mice

**DOI:** 10.1371/journal.pone.0150682

**Published:** 2016-02-26

**Authors:** Raffaella Tulino, Agnesska C. Benjamin, Nelly Jolinon, Donna L. Smith, Eduardo N. Chini, Alisia Carnemolla, Gillian P. Bates

## Notice of Republication

This article was republished on February 19, 2016, to correct errors in Fig 2 and Fig 6. Fig 2 was replaced with an incorrect version of Fig 3 and an enlarged version of Fig 6 was published in the original article. Please download this article again to view the correct version. The originally published, uncorrected article and the republished, corrected articles are provided here for reference.

## Supporting Information

S1 File Originally published, uncorrected article. (PDF)

S2 File Republished, corrected article. (PDF)
