# Supplementary figures and images for: SIRT1 Activity Is Linked to Its Brain Region-Specific Phosphorylation and Is Impaired in Huntington’s Disease Mice
Source: PLoS One. 2016 Jan 27;11(1):e0145425. doi: 10.1371/journal.pone.0145425 (PMC4731418; doi:10.1371/journal.pone.0145425)

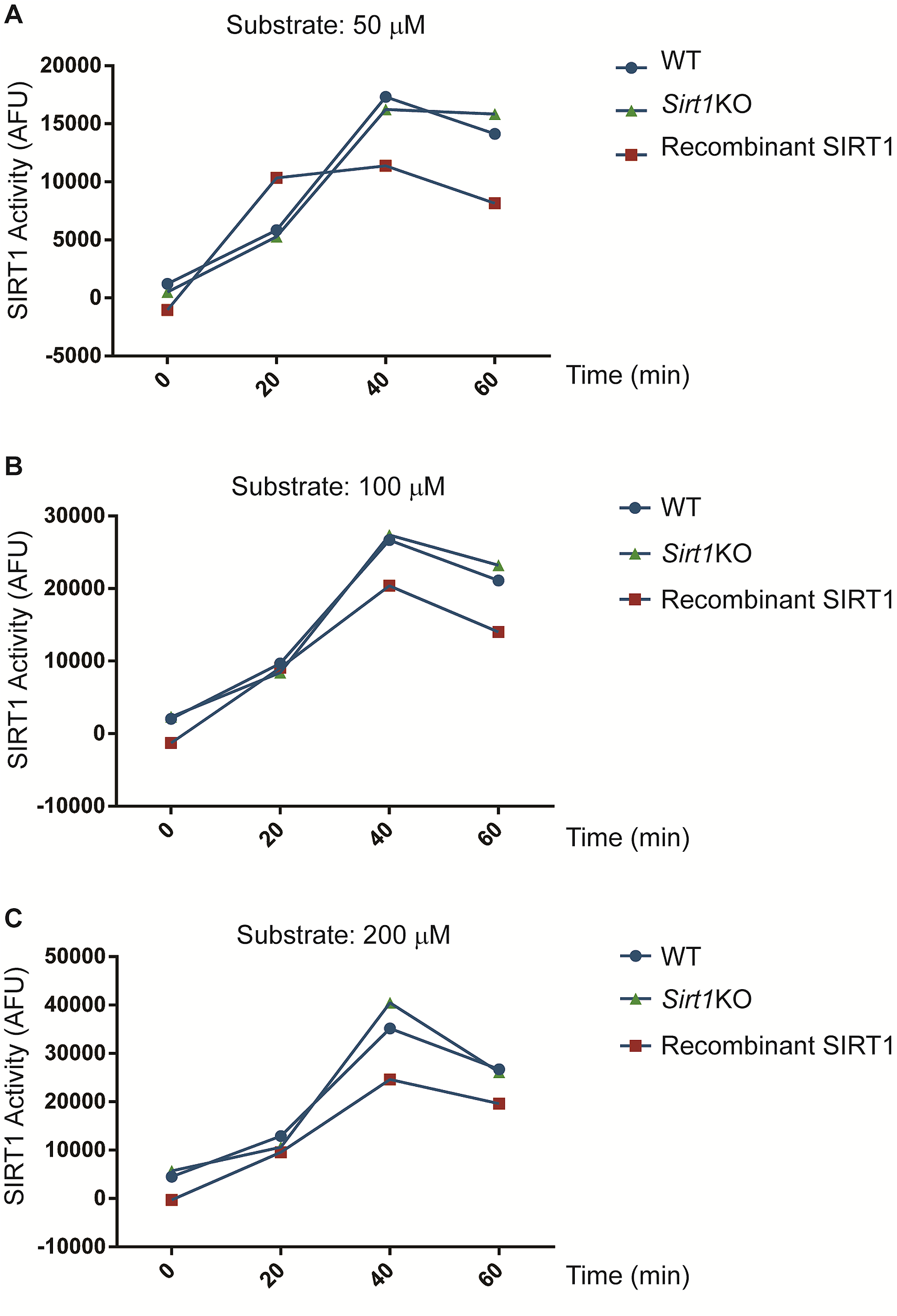

Supplement: S1 Fig — Fluorescence signal obtained after 0, 20, 40 and 60 minute incubations at 37°C. 25 μg of protein from cortical lysates of WT or Sirt1KO mice and 2U/well of SIRT1-recombinant protein were incubated with (A) 50, (B) 100 or (C) 200 μM of substrate and with 200 μM of NAD+ and 5 μM TSA. n = 2 / genotype. AFU = arbitrary fluorescence units. (TIF) [file pone.0145425.s002.tif]

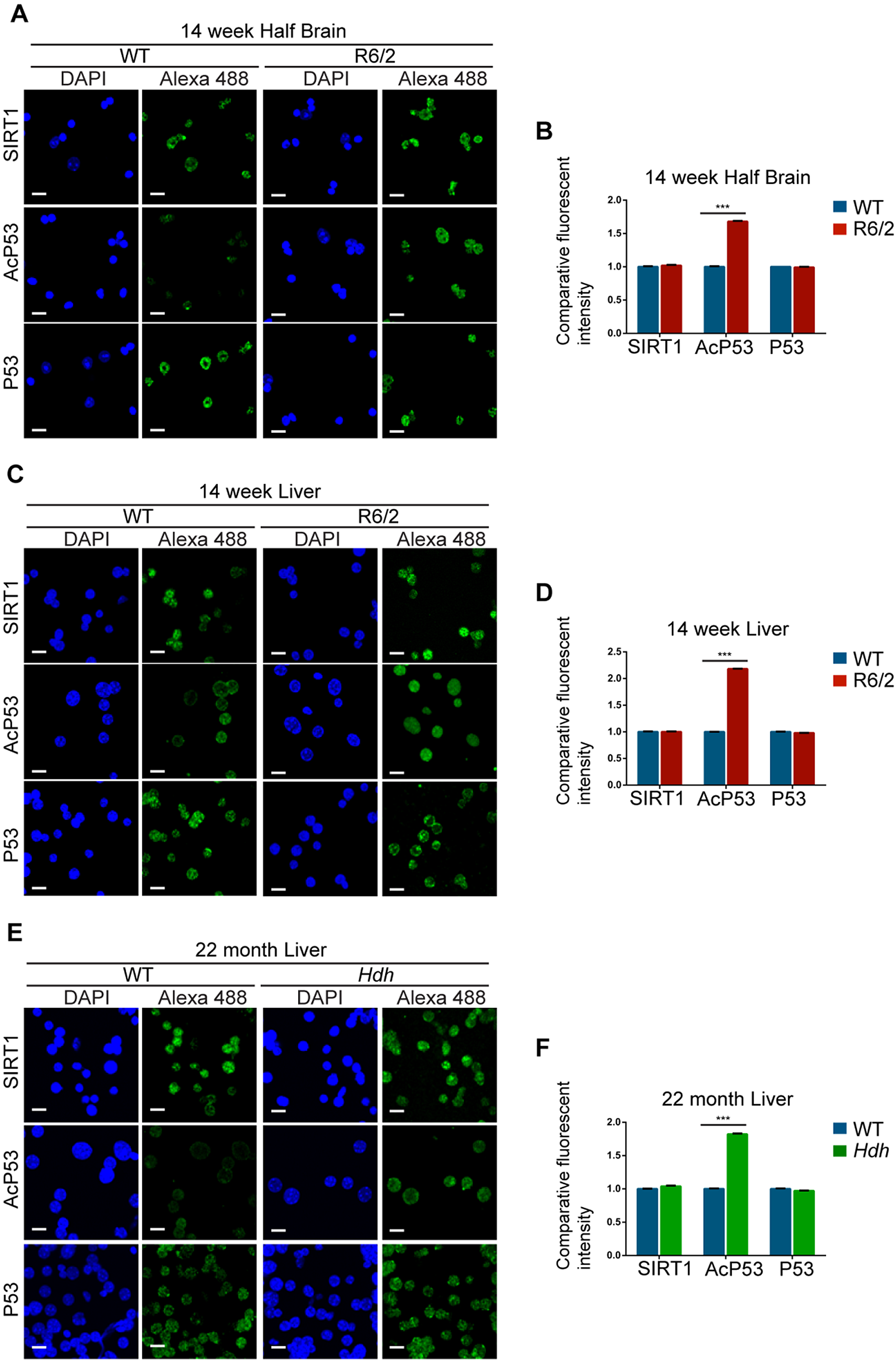

Supplement: S2 Fig — Representative immunofluorescence image of isolated nuclei extracted from (A) half brain and (C) liver from R6/2 mice at 14 weeks of age and (E) liver from 22 month HdhQ150 mice immunostained for SIRT1, P53 and AcP53 and counterstained with DAPI. (B, D, F) Relative intensity levels of SIRT1, p53 and Acp53 from immunostained nuclei in (A, C, E) respectively. The quantification indicates that the level of acetylated P53 is higher in the HD models, consistent with a decrease in SIRT1 activity as depicted in Fig 2C. Scale bar, 10 μm. n = 4 / genotype. (TIF) [file pone.0145425.s003.tif]

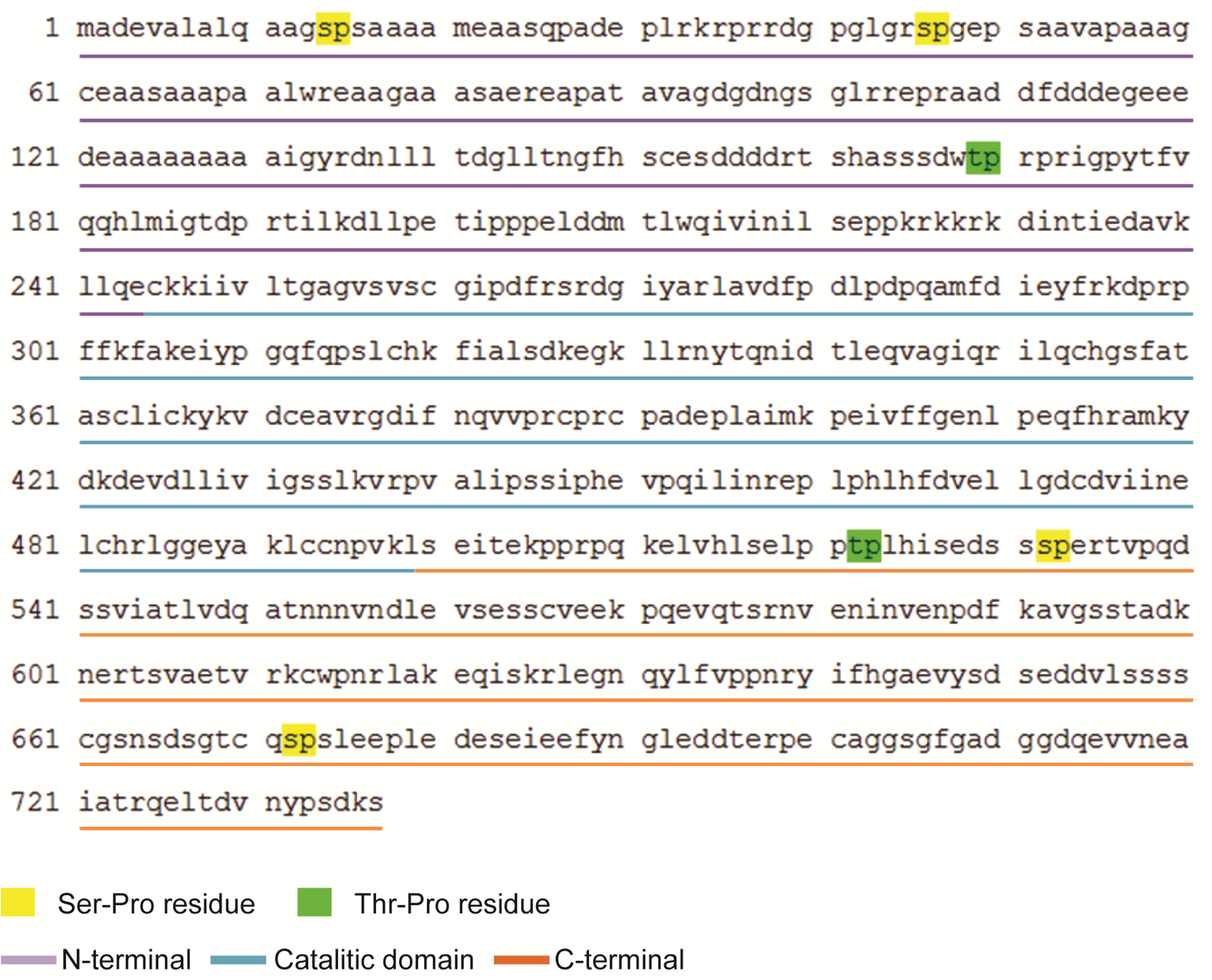

Supplement: S3 Fig — Ser-Pro residues are highlight in yellow and Thr-Pro residues are highlight in green. Notably, all the phosphorylation sites recognized by the Mpm2 antibody are located in the N-terminal (1-244aa, purple) and C-terminal (498-737aa, orange) regions. The catalytic domain of SIRT1 is underlined in blue. (TIF) [file pone.0145425.s004.tif]

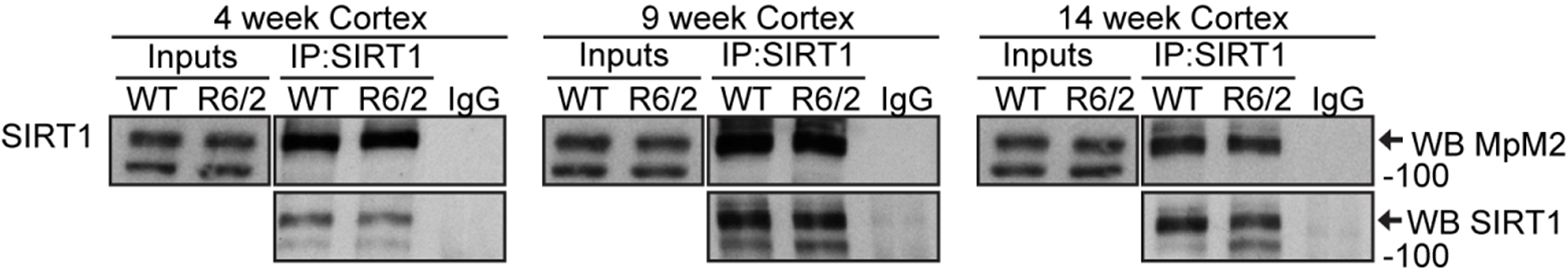

Supplement: S4 Fig — Western blots of SIRT1 and phosphorylated SIRT1 (MpM2) after SIRT1 immunoprecipitation from cortical lysates of R6/2 mice at 4, 9 and 14 weeks of age compared to WT littermates. IP = immunoprecipitation, WB = western blotting. (TIF) [file pone.0145425.s005.tif]

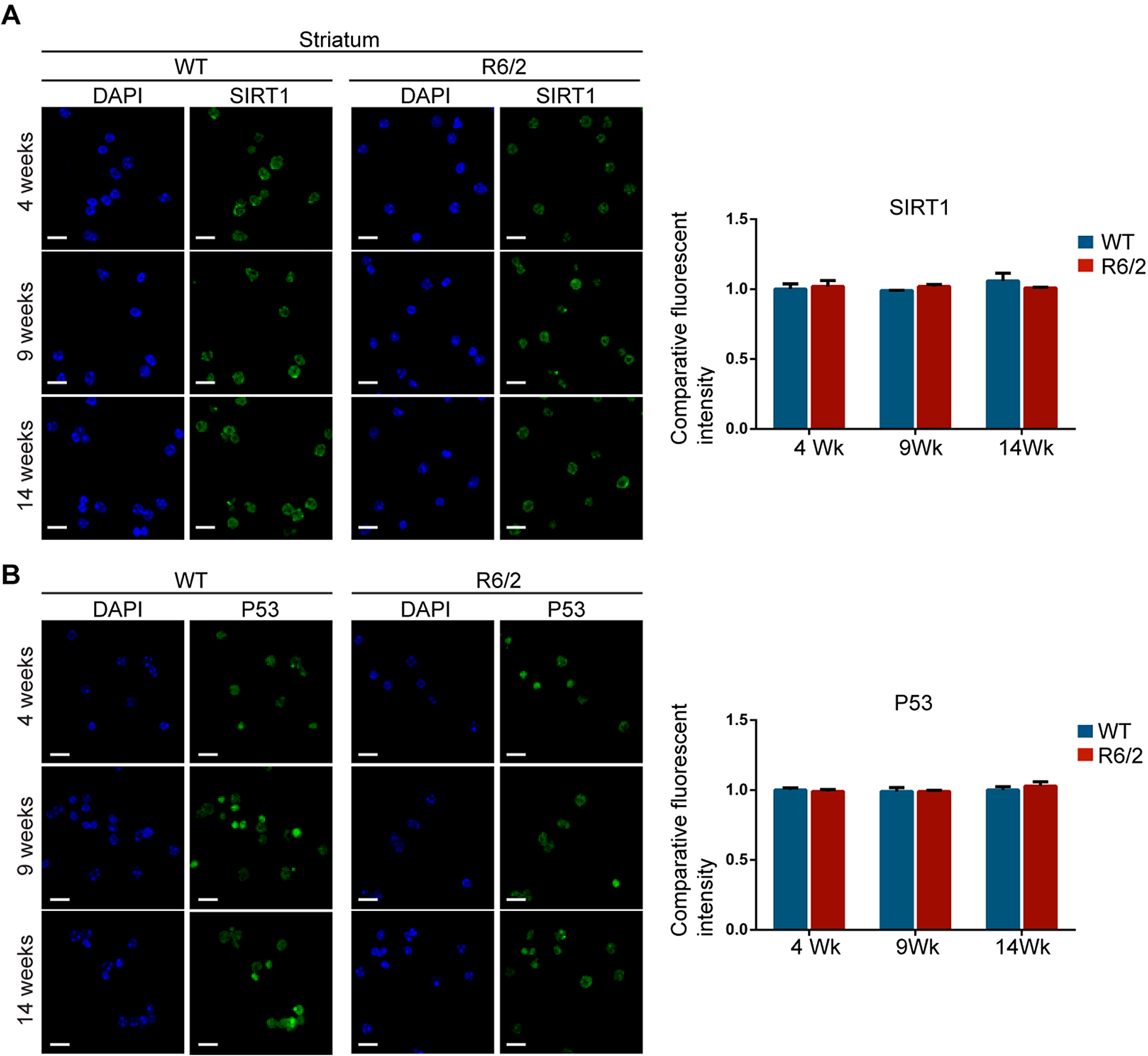

Supplement: S5 Fig — Representative immunofluorescence images of isolated nuclei extracted from the striata of WT and R6/2 mice at 4, 9 and 14 weeks of age immunostained for (A) SIRT1 and (B) P53, and counterstained with DAPI. The relative intensity levels of SIRT1 and P53 are depicted alongside. Scale bar, 10 μm. Data are the mean ± SEM. n = 10 / genotype in 2 pools of 5 striata. (TIF) [file pone.0145425.s006.tif]

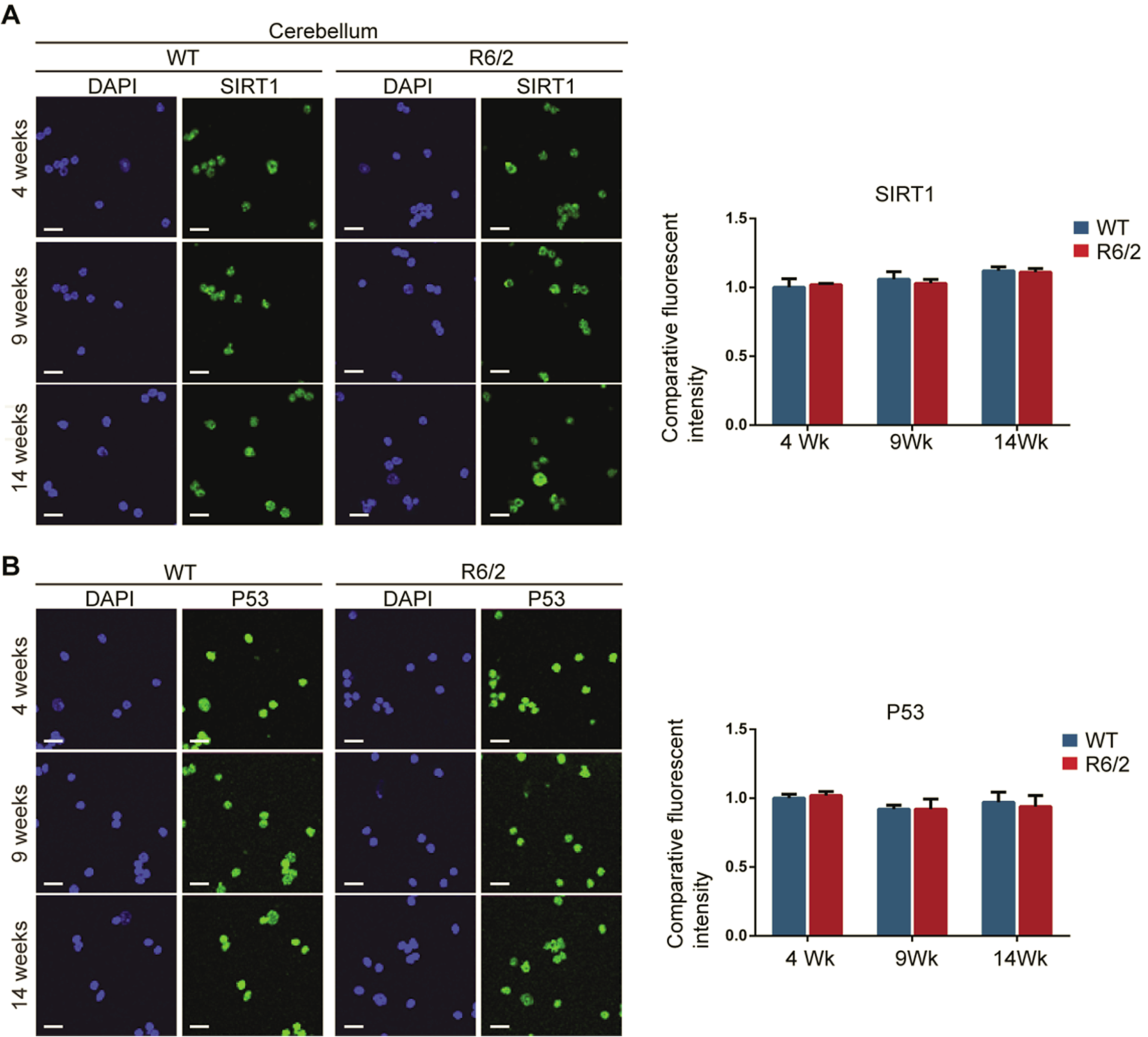

Supplement: S6 Fig — Representative immunofluorescence image of isolated nuclei extracted from the cerebella of WT and R6/2 mice at 4, 9 and 14 weeks of age immunostained for (A) SIRT1 and (B) P53, and counterstained with DAPI. The relative intensity levels of SIRT1 and P53 are depicted alongside. Scale bar, 10 μm. Data are the mean ± SEM. n = 10 / genotype in 2 pools of 5 cerebella. (TIF) [file pone.0145425.s007.tif]

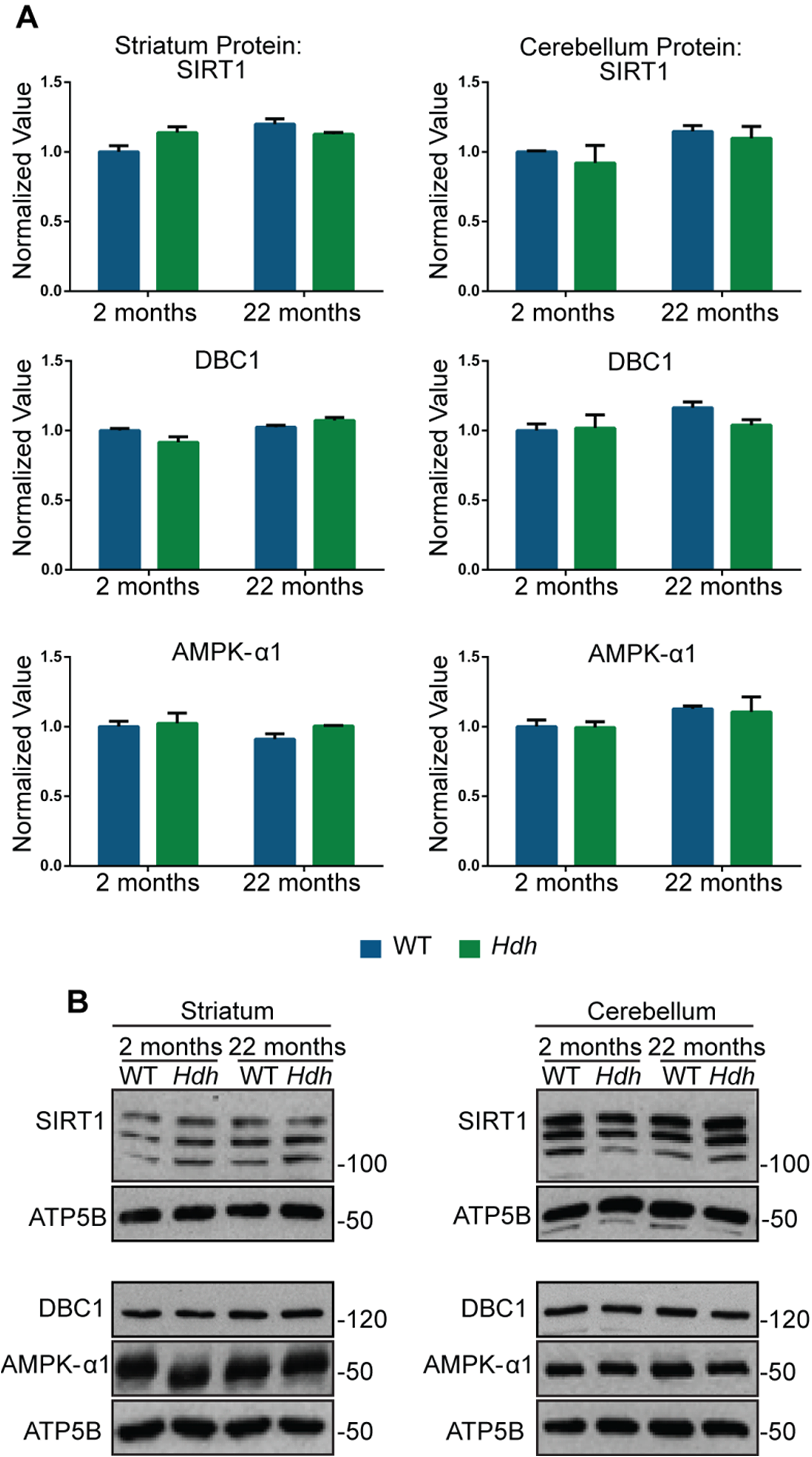

Supplement: S7 Fig — (A) Relative protein levels and (B) representative western blots of SIRT1, DBC1 and AMPK-α1 in striatum and cerebellum of 2 and 22-month HdhQ150 homozygotes and WT mice. Densitometric values were calculated relative to 2-month WT mice. Data are mean ± SEM. Hdh = HdhQ150 homozygotes. n = 4 / genotype. (TIF) [file pone.0145425.s008.tif]

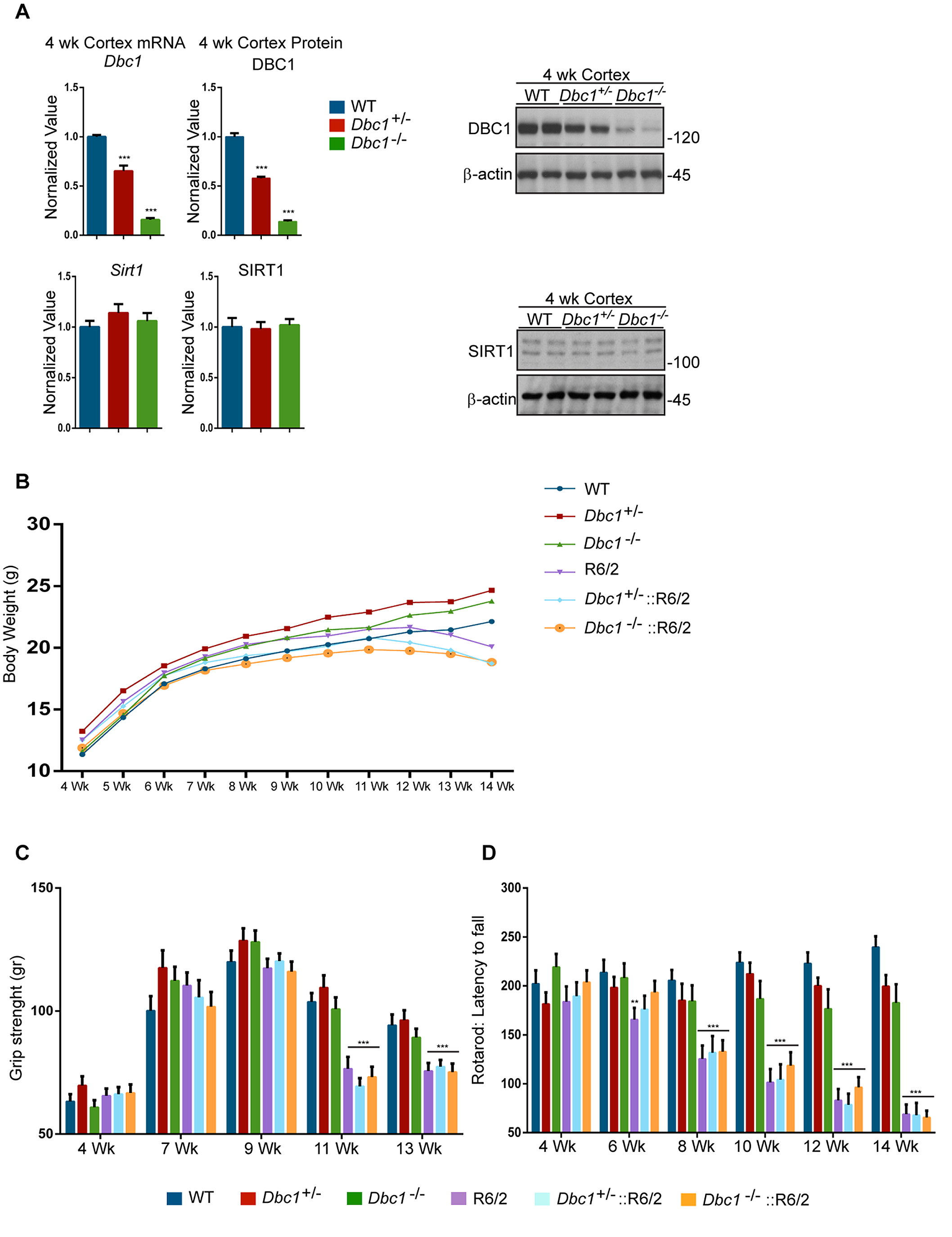

Supplement: S8 Fig — (A) Analysis of DBC1 and SIRT1 expression in 4-week cortex of WT, Dbc1+/- and Dbc1-/- mice. (B) Body weight, (C) forelimb grip strength and (D) latency to fall from the rotarod were measured from 4 to 14 weeks of age. Number of mice: WT: 22 (7M-15F), Dbc1+/-: 19 (11M-8F), Dbc1-/-: 18 (9M-9F), R6/2: 22 (13M-9F), Dbc1+/-::R6/2: 18 (9M-9F), Dbc1-/-::R6/2: 19 (6M-13F). Data are the mean ± SEM. ***P<0.001: statistically significant difference as compared to WT. (TIF) [file pone.0145425.s009.tif]
