# Supplementary material for: SIRT1 Activity Is Linked to Its Brain Region-Specific Phosphorylation and Is Impaired in Huntington’s Disease Mice
Source: PLoS One. 2016 Jan 27;11(1):e0145425. doi: 10.1371/journal.pone.0145425 (PMC4731418; doi:10.1371/journal.pone.0145425)
Supplement: S1 Table — (DOCX) [file pone.0145425.s010.docx]

**S1 Table. Summary of the working dilution and application of all antibodies.**

| Antibody | Catalogue # | Source | Dilution |
| --- | --- | --- | --- |
| DBC1 | A300-432A | Bethyl lab. | 1:1000 (WB)  1:100 (IHC) |
| SIRT1 | ab12193 | Abcam | 1:1000 (WB)  1:100 (IHC) |
| P53 | sc-6243 | Santa cruz | 1:100 (IHC) |
| AcP53 | ab122899 | Abcam | 1:50 (IHC) |
| MpM2 | 05-368 | Millipore | 1:1000 (WB) |
| HTT | MAB5374(EM48) | Millipore | 1:100 (IHC) |
| HTT | MAB2166 | Millipore | 1:2500 (WB) |
| AMPKα1 | A300-507A | Bethyl lab. | 1:500 (WB) |
| AMPKα1 | ab32047 | Abcam | 1:10 (IP) |
| β-actin | sc-47778 | Santa-Cruz | 1: 10000 (WB) |
| ATP5B | ab14730 | Abcam | 1:15000 (WB) |
| α-tubulin | T9026 | Sigma | 1:30000 (WB) |
| Histone H3 | ab1791 | Abcam | 1:30000 (WB) |

WB = western blotting; IP = immunoprecipitation; IHC = immunohistochemistry.
