# Supplementary material for: SIRT1 Activity Is Linked to Its Brain Region-Specific Phosphorylation and Is Impaired in Huntington’s Disease Mice
Source: PLoS One. 2016 Jan 27;11(1):e0145425. doi: 10.1371/journal.pone.0145425 (PMC4731418; doi:10.1371/journal.pone.0145425)
Supplement: S2 Table — (DOCX) [file pone.0145425.s011.docx]

**S2 Table.** **Summary of in-house designed primer and probe sequences for qPCR.**

| Name | Application | 5'-3' sequence |
| --- | --- | --- |
| *Dbc1 forward* | RT-qPCR | GTACCAGAAGGCAGCTGGAG |
| *Dbc1 reverse* | RT-qPCR | TGAAGAGGCTGAGCACAGAA |
| *Dbc1 probe* | RT-qPCR | AGGCTACCCCTGCTGTGGCTCCCA |
| *Sirt1 forward* | RT-qPCR | TGTTGGTTGACTTCATCTTCCTT |
| *Sirt1 reverse* | RT-qPCR | TCCAATGGCTTTTGAAAACTTTA |
| *Sirt1 probe* | RT-qPCR | TTCATTTGTATGATACATTCGTATGTATG |
| *Ampk-α1* | RT-qPCR | Assay was purchased from ABI |

All RT-qPCR probes were labelled 5' with FAM and 3' with TAMRA. RT-qPCR real time-quantitative PCR*.*
