# Supplementary material for: Correction: SIRT1 Activity Is Linked to Its Brain Region-Specific Phosphorylation and Is Impaired in Huntington's Disease Mice
Source: PLoS One. 2016 Feb 26;11(2):e0150682. doi: 10.1371/journal.pone.0150682 (PMC4769183; doi:10.1371/journal.pone.0150682)
Supplement: S1 File — (PDF) [file pone.0150682.s001.pdf]

RESEARCH ARTICLE

SIRT1 Activity Is Linked to Its Brain Region-Specific Phosphorylation and Is Impaired in Huntington's Disease Mice

Raffaella Tulino[1], Agnesska C. Benjamin[1], Nelly Jolinon[1], Donna L. Smith[1], Eduardo N. Chini[2], Alisia Carnemolla[1]*, Gillian P. Bates[1]*

1 Department of Medical and Molecular Genetics, King's College London, London, United Kingdom, **2** Mayo Clinic College of Medicine, Rochester, Minnesota, United States of America

* gillian.bates@kcl.ac.uk (GPB); alisia.carnemolla@kcl.ac.uk (AC)

CrossMark
click for updates

OPEN ACCESS

Citation: Tulino R, Benjamin AC, Jolinon N, Smith DL, Chini EN, Carnemolla A, et al. (2016) SIRT1 Activity Is Linked to Its Brain Region-Specific Phosphorylation and Is Impaired in Huntington's Disease Mice. PLoS ONE 11(1): e0145425. doi:10.1371/journal.pone.0145425

Editor: Xiao-Jiang Li, Emory University, UNITED STATES

Received: October 8, 2015

Accepted: November 2, 2015

Published: January 27, 2016

Copyright: © 2016 Tulino et al. This is an open access article distributed under the terms of the Creative Commons Attribution License, which permits unrestricted use, distribution, and reproduction in any medium, provided the original author and source are credited.

Data Availability Statement: All relevant data are within the paper and its Supporting Information files.

Funding: RT was funded by a Marie Curie Training Network from the European Community Program FP7/2010 under grant agreement no. 264508 and this funder had no role in study design, data collection and analysis, decision to publish, or preparation of the manuscript. This work was also supported by the CHDI Foundation, a not-for-profit biomedical research organization exclusively dedicated to discovering and developing therapeutics that slow the progression of Huntington's disease. Some of this research

Abstract

Huntington's disease (HD) is a neurodegenerative disorder for which there are no disease-modifying treatments. SIRT1 is a NAD^+-dependent protein deacetylase that is implicated in maintaining neuronal health during development, differentiation and ageing. Previous studies suggested that the modulation of SIRT1 activity is neuroprotective in HD mouse models, however, the mechanisms controlling SIRT1 activity are unknown. We have identified a striatum-specific phosphorylation-dependent regulatory mechanism of SIRT1 induction under normal physiological conditions, which is impaired in HD. We demonstrate that SIRT1 activity is down-regulated in the brains of two complementary HD mouse models, which correlated with altered SIRT1 phosphorylation levels. This SIRT1 impairment could not be rescued by the ablation of DBC1, a negative regulator of SIRT1, but was linked to changes in the sub-cellular distribution of AMPK-α1, a positive regulator of SIRT1 function. This work provides insights into the regulation of SIRT1 activity with the potential for the development of novel therapeutic strategies.

Introduction

Huntington's disease (HD) is a devastating neurodegenerative disorder caused by a CAG repeat expansion within exon 1 of the huntingtin gene (*HTT*), which encodes for an expanded polyglutamine (polyQ) tract in the huntingtin protein (HTT) [1]. Symptoms usually appear in mid-life, comprise personality changes, problems with motor coordination and cognitive decline; disease duration lasts between 15 and 20 years and there are no disease-modifying treatments [2]. The neuropathology of HD is characterised by neuronal cell death in the striatum, cortex and other brain regions and the accumulation of cytoplasmic and nuclear aggregates [3].

Mouse models of HD include those that are transgenic for N-terminal fragments of HTT (e.g. R6/2) or the full length HTT protein or are knock-in models in which the HD mutation has been introduced into mouse *Htt* (e.g. *Hdh*Q150) [4]. The R6/2 mouse is transgenic for an exon 1 HTT protein [5] and is a model of the aberrant splicing that occurs in HD [6]. The

conducted at King's College London was performed in collaboration with and funded by the CHDI Foundation.

Competing Interests: The authors declare that there are no patents, products in development or marketed products to declare. The authors fully adhere to all the PLOS ONE policies on sharing data and materials, as detailed online in the guide for authors.

*Hdh*Q150 model had a 150 CAG repeat knocked into the mouse *Htt* gene [7]. In addition to the full length protein, *Hdh*Q150 mice express mutant exon 1 HTT through aberrant splicing [6] and many other N-terminal HTT fragments generated through proteolysis [8]. At late stage disease (14 weeks for R6/2 and 22 months for homozygous *Hdh*Q150 mice) these models exhibit remarkably similar phenotypes [9–14] the main difference between these two models being the age of disease onset and rate of disease progression.

SIRT1, a mammalian orthologue of the yeast Sir2 protein, is a NAD^{+}-dependent deacetylase that plays a critical role in multiple biological processes including apoptosis [15], ageing [16], metabolism [17] and various stress responses [18]. It has been demonstrated that DBC1 (deleted in breast cancer 1) inhibits SIRT1 via a direct interaction with its catalytic domain [19]. This dynamic interaction is sensitive to the energetic state of the cell and involves the activity of AMPK (AMP-activated protein kinase), an important cellular energy sensor [20]. In circumstances of low cellular energy, AMPK stimulates compensatory processes, including the activation of SIRT1, resulting in the restoration of ATP levels [21]. However, the complexity of SIRT1 functions in the mammalian brain and the mechanisms involved in SIRT1 regulation are not fully understood.

SIRT1 has been shown to participate in neuronal protection and survival in various mouse models of neurodegenerative disorders through a number of substrates such as P53 [22] and HSF1 [23]. With relevance to HD, the activation of Sir2 was protective against mutant phenotypes in a *C. elegans* model [24]. Increased expression of *Sirt1* attenuated neurodegeneration and improved motor function in N171-82Q and BACHD mice [25] and attenuated brain atrophy and reduced mutant HTT aggregation in R6/2 mice without prolonging lifespan [26]. More recently, SRT2104, a SIRT1 activator was reported to have beneficial effects in an HD mouse model [27] with the potential for interrogating SIRT1 activity in the clinic [28]. In contrast, a SIRT1 inhibitor, selisistat, has been reported to alleviate HD-related phenotypes in multiple HD models [29] and has been found to be safe in clinical trials [30]. Based on these findings, the mis-regulation of SIRT1 could have important implications in the development and progression of HD.

In this study we describe a striatum-specific phosphorylation-dependent regulatory mechanism that controls SIRT1 activity under normal physiological conditions that is impaired in HD. We show that SIRT1 activity is decreased in the brains of R6/2 and *Hdh*Q150 mice, and that this is not caused by the sequestration of SIRT1 into HTT inclusions. We demonstrate that the presence of mutant HTT in the striatum and cerebellum of HD mice alters the phosphorylation status of SIRT1 and that these effects are related to the abnormal expression and cellular localization of AMPK-α1. Finally, we show that the ablation of DBC1, a negative regulator of SIRT1 [31] does not rescue the deficit in SIRT1 activity in HD mouse models. These results provide new insights into the mechanisms that regulate SIRT1 function and may lead to the development of new strategies by which SIRT1 can be manipulated for therapeutic benefit.

Materials and Methods

Ethics

All experimental procedures performed on mice were conducted under a project licence from the Home Office (Animal Scientific Procedures Act 1996) and approved by the King's College London Ethical Review Process Committee.

Mouse maintenance, breeding, genotyping, and CAG repeat sizing

Hemizygous R6/2 mice [5] were bred by backcrossing R6/2 males to (CBA × C57BL/6) F1 females (B6CBAF1/OlaHsd; Harlan Olac). $Hdh^{Q150/Q150}$ homozygous mice [9] on a

(CBA × C57BL/6) F1 background were generated by intercrossing *Hdh*$^{Q150/Q7}$ heterozygous CBA/Ca and C57BL/6J congenic lines (inbred lines from Harlan Olac). R6/2 and *Hdh*$^{Q150/Q150}$ homozygous mice were genotyped and the CAG repeat was sized as previously described [32]. The mean repeat size (± SD) for all mice used in the entire study was 165 ± 10 for *Hdh*$^{Q150/Q150}$ homozygous mice and 204 ± 7 for R6/2 mice. *Dbc1* heterozygous mice were obtained from the Eduardo Chini at the Mayo Foundation, Mayo Clinic College of Medicine, Rochester, Minnesota, USA. PCR conditions for genotyping *Dbc1* knock-out mice have been previously described [19]. *SirT1* floxed homozygous (SirT1 Fl/Fl) mice were obtained from the JAX Laboratory (Mouse Strain: B6;129-SirT1tm1Ygu/J) [33] and were bred with β-actin/Cre heterozygous mice to generate complete *Sirt1*KO mice. *Sirt1* transgenic mice (CBA×C57BL/6J) [34] were obtained from David Holzman's laboratory at Washington University, Missouri, USA

Animals were housed under 12 h light/12 h dark cycle, with unlimited access to water and food (Special Diet Service, Witham, UK) in a conventional Unit. Cages were environmentally enriched with a cardboard tube. R6/2 mice and all mice in phenotypic assessment trials were always given mash food consisting of powered chow mixed with water from 12 weeks of age until sacrificed. Upon sacrifice, dissected brain regions, whole brains or peripheral tissues were snap frozen in liquid nitrogen and stored at -80°C until use.

Mouse behavioural analysis

At 4 weeks of age, mice were weaned into cages of 5–6 animals. Each cage contained at least one representative of each genotype from mixed litters. The analysis of mice of different genotypes was distributed equally throughout the assessment period on any given day and all behavioural tests were performed blind to the investigator. Mice were weighed weekly and rotarod performance and grip strength were assessed as previously reported [35–37]. The statistical power of these tests was calculated as previously described [37]. The data were analysed by repeated measures general linear model ANOVA using SPSS software [37].

Protein extraction for SDS PAGE, Immunoblotting and Immunoprecipitation

Frozen mouse brain tissue was homogenized in 1 volume of ice cold NETN buffer (20 mM Tris-HCl pH 8, 100 mM NaCl, 1 mM EDTA, 0.5% NP-40, complete protease inhibitors and phosphatase inhibitors) using a polytron homogenizing probe. Samples were sonicated on ice with a vibracell sonicator (10 x 1 s 20 kHz pulses) and spun at 13,000 x *g* for 10 min at 4°C. The supernatant was retained and protein concentration was determined for each sample by the BCA assay (Thermo Scientific).

SDS PAGE and Immunoblotting

Protein lysates were diluted with 2x Leammli Buffer, denatured for 10 min at 95°C, loaded onto SDS polyacrylamide gels and subjected to western blot as previously described [8]. Membranes were blocked in 5% non-fat dried milk in PBS—0.2% Tween 20 (PBS-T) or 4% BSA for 2 h at RT. Primary antibodies were added overnight at 4°C in 5% non-fat dried milk in PBS-T (DBC1, SIRT1, HTT, AMPK-α1,) or 4% BSA (MpM2). β-actin, ATP5B, α-tubulin and histone pan-H3 were incubated for 20 min at RT in 5% non-fat dried milk in PBS-T. Blots were washed three times for 10 min in 0.2% PBS-T, incubated with the appropriate secondary antibody for 1 h at RT, washed three times for 10 min in 0.2% PBS-T and exposed to ECL according to manufacturer's instruction (Amersham). The signal was developed using Amersham hyperfilm and Xenograph developer. Densitometry of western blots was performed using a Bio-Rad GS-800 densitometer. Developed films were scanned and the average pixel optical density (OD) for

each band was measured using QuantityONE software. The OD of an area devoid of bands was subtracted from the values obtained for bands of interest in order to normalize the OD against background. Relative expression was determined by dividing the normalized OD of bands of interest by the OD of the appropriate loading control for each sample. For full details of primary antibodies see S1 Table.

Immunoprecipitation

Protein lysates were prepared for immunoprecipitation (IP) as described above. For IP from striatal lysates, striata were pooled from two animals. IP reactions were performed in 1 ml of NETN buffer containing from 400 to 1000 μg protein and 1 μg of antibody and normal rabbit IgG (#2729; Cell Signaling) was used as a negative control. Reactions were left on a rotating wheel at 4°C for 90 min (AMPK-α1) or 4 h (SIRT1) and 15 μl of protein G-coupled Dynabeads (10004D; Life Technologies) were added for the last 45 min. Following IP, protein G-coupled Dynabeads were briefly spun at 13,000 x *g* for 30 sec, put on a magnetic rack, washed with 1 ml of NETN buffer (4x) and re-suspended in 15 μl of 2x Leammli buffer. Immuno-precipitated complexes were eluted from the beads by denaturation at 100°C for 10 min and immediately loaded for SDS-PAGE analysis.

Nuclear/cytoplasmic fractionation

All steps were performed on ice. Half brain tissue or liver was cut into small pieces and homogenized with a Dounce homogenizer in TKM buffer (0.25 M sucrose; 50 mM Tris-HCl, pH 7.4; 25 mM KCl; 5 mM MgCl2 and 1 mM PMSF) and nuclear and cytoplasmic fractions were prepared as previously described [19]. For the nuclear and cytoplasmic preparations from brain regions, striata were pooled from four animals, whereas a single cerebellum was used. The final pellet containing the purified nuclei was resuspended in 4% PFA for immunohistochemistry or in NETN buffer for protein analysis and protein concentration was determined by the BCA assay (Thermo Scientific).

Immunohistochemistry

The isolation of nuclei from brain or liver was as described above. Nuclei were extracted from 4 mice per genotype from half brain or liver and for brain regions, from two pools each containing specific regions from five mice. Samples were fixed on the slide for 30 min with 4% paraformaldehyde prepared in PBS, permeabilized with 0.1% Triton X-100 in PBS for 15 min, washed 3X with PBS, and incubated for 1 h at RT in blocking buffer (PBS with 0.1% Triton and 1% BSA). Nuclei were incubated with the primary antibody in blocking buffer (DBC1, SIRT1, P53 and Ac-P53) overnight at 4°C, washed 3x with PBS at RT and then incubated with the secondary antibody and DAPI in PBS—0.1% Triton for 1 h at RT. Samples were mounted using VECTASHIELD mounting medium. Nuclei were visualized using a TCS SP2 Leica confocal microscope. Fluorescence intensity was quantified from 50 nuclei per sample imaged from 10 fields of view per slide using ImageJ. Ac-P53 levels were normalised to the P53 intensity level. Fluorescent intensity levels were presented as a fold change from WT levels as indicated in the figures. The direction of the fold change was inverted to depict the comparative deacetylase activity.

Fluor de Lys assay

SIRT1 activity was determined with a SIRT1 Fluorometric Kit (BML-AK555) according to the manufacturer's instructions. Protein extraction was performed as described above. Homogenates were then incubated for 10 min at 37°C to allow degradation of any contaminant NAD^+.

10 mM DTT was added to the medium, and homogenates were incubated again for 10 min at 37°C. The homogenates (20–30 μg protein/well) were then incubated in SIRT1 assay buffer in the presence of 50, 100 or 200 μM Fluor de Lys–SIRT1 substrate (Enzo Life Sciences), 5 μM TSA and 200 μM NAD^+. After 0-, 20-, 40- and 60 minutes of incubation at 37°C, the reaction was terminated by adding a solution containing Fluor de Lys Developer (Enzo Life Sciences) and 2 mM nicotinamide. After 1 h the values were determined by reading fluorescence on a fluorometric plate reader (Spectramax Gemini XPS; Molecular Devices) with an excitation wavelength of 360 nm and an emission wavelength of 460 nm.

Taqman RT-qPCR

RNA extraction, cDNA sysnthesis, Taqman RT-qPCR and ΔCt analysis were performed as described previously [38]. The Taqman qPCR assays were purchased from Primer Design and ABI. For a list of primers and probes, see S2 Table.

Statistical Analysis

Statistical analysis was performed with SPSS (repeated measures ANOVA General Linear Model) or Microsoft Excel (Student's *t*-test) software. *p*-values of <0.05 were considered significant. Graphs were constructed using Prism Ver.5.0b (GraphPad Software).

Results

SIRT1 function becomes compromised in the brains of HD mice

There is considerable evidence to support the beneficial effect of SIRT1 manipulation in HD mouse models. However, the impact of mutant HTT on SIRT1 function has not been fully elucidated. As such, we set out to analyse SIRT1 activity and the mechanisms involved in its regulation in two different mouse models of HD: R6/2 transgenic and *Hdh*Q150 knock-in homozygous mice.

SIRT1 regulates the activity of several transcription factors including P53 [39]. It deacetylates P53 on Lys382 thereby inhibiting its function [40]. There are a number of commercial kits that use the deacetylation of this P53 lysine residue to assess SIRT 1 activity. In order to have a direct measurement of SIRT1 activity, we applied the Fluor-de-Lys fluorometric activity assay (Enzo Laboratories). The specificity of the kit was evaluated on lysates from the brains of SIRT1 knock-out (*Sirt1*KO) [33] mice at 4 weeks of age, but unfortunately we found that this kit was not specific for SIRT1 in these brain lysates (S1 Fig). Therefore we tested an alternative published method to assess the steady-state levels of SIRT1 activity on endogenous P53 in mouse brains that makes use of nuclei purified from mouse tissues [19]. The genotypes of the mice used for the experiment were verified by western blot (Fig 1A). Nuclei were isolated from the brains of *Sirt1*KO and *Sirt1*Tg mice [34] at 4 weeks of age and immunostained for P53 and acetylated-P53 (AcP53) at Lys382, and counterstained with DAPI (Fig 1B). P53 levels were equivalent between the *Sirt1*KO and *Sirt1*Tg lines and the corresponding wild type (WT) littermates (Fig 1C). The acetylation of P53 Lys 382 was considerably increased in the *Sirt1*KO nuclei and decreased in those from the *Sirt1*Tg mice, consistent with a decrease in SIRT1 activity in the knock-out line and an increase in SIRT1 activity in the transgenic line respectively (Fig 1C), demonstrating that this approach could be used to monitor the steady-state level of SIRT1 activity in mouse brain.

To monitor the level of SIRT1 activity in HD mouse models, we isolated cell nuclei from the brains of R6/2 mice at 4, 9 and 14 weeks of age and *Hdh*Q150 homozygous mice at 2 and 22 months together with their aged-matched WT littermates. Nuclei were immunostained for

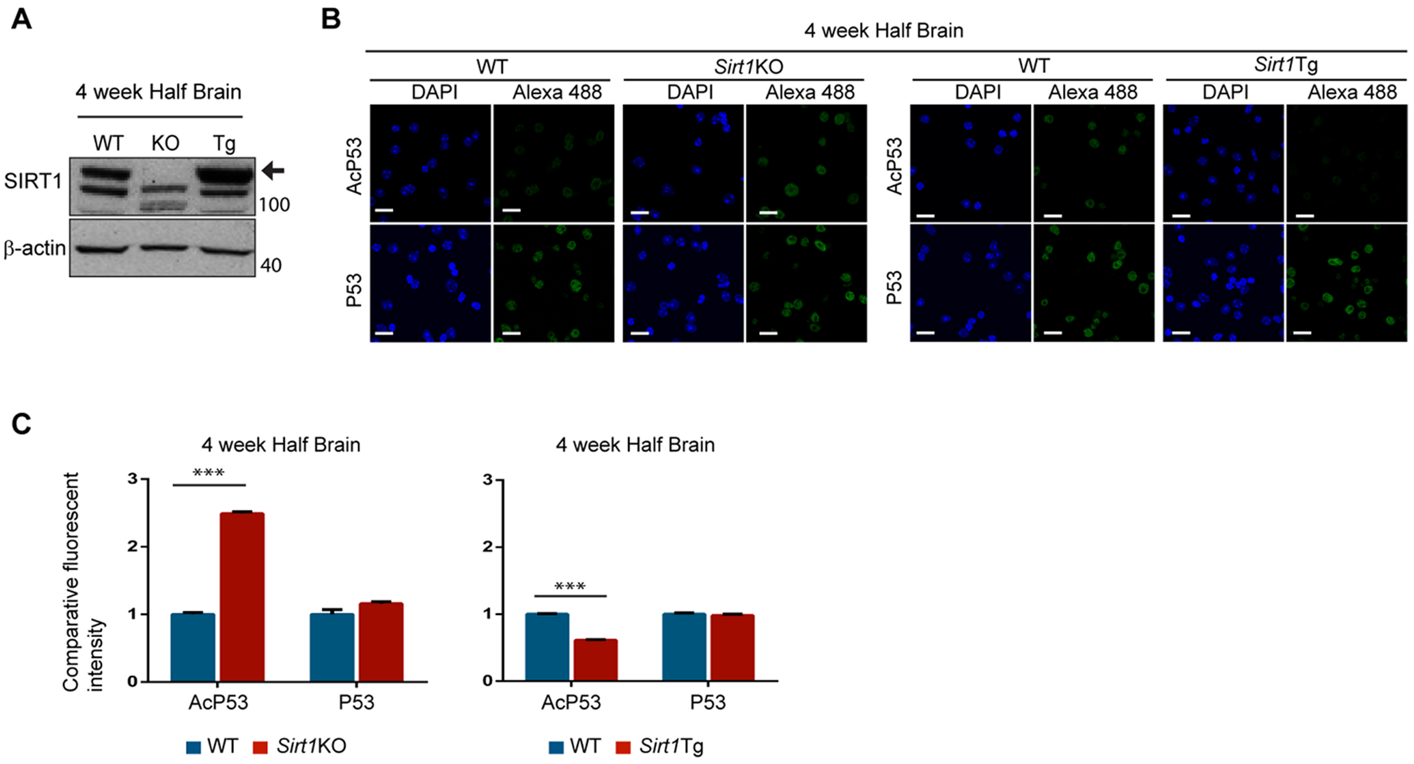

Fig 1. Method for monitoring the steady-state levels of SIRT1 activity in mouse tissues. (A) Representative western blot for SIRT1 in half brain from *Sirt1*KO and *Sirt1*Tg mice at 4 weeks of age. (B) Representative immunofluorescence image of isolated nuclei extracted from half brains from *Sirt1*KO and *Sirt1*Tg mice at 4 weeks of age immunostained for P53 and AcP53 and counterstained with DAPI. (C) Relative intensity level of P53 and AcP53 from the immunostained nuclei shown in B. The quantification indicates that the level of acetylated P53 is higher in the *Sirt1*KO mice, consistent with a decrease in SIRT1 activity, and lower in *Sirt1*Tg mice, consistent with an increase in SIRT1 activity. Data are the mean ± SEM. ***$p<0.001$. Scale bar, 10 μm; KO = *Sirt1*KO; Tg = *Sirt1*Tg. n = 4 / genotype.

doi:10.1371/journal.pone.0145425.g001

SIRT1, P53 and acetylated-P53 (AcP53), and counterstained with DAPI (Fig 2A and S2A Fig). We did not detect any variation in the intensity level of SIRT1 and P53 staining between HD mouse samples and their corresponding WT controls at each age of analysis (Fig 2B and S2B Fig). In contrast, whilst we found that the acetylation levels of endogenous P53 were equivalent in HD as compared to WT littermate brains in presymptomatic mice (i.e. 4 week R6/2 and 2 month *Hdh*Q150 homozygotes) (Fig 2A and 2B), the level of AcP53 was significantly higher (≥ 1.5 fold) in samples from early symptomatic R6/2 mice (9 weeks) and late stage symptomatic R6/2 (14 weeks) and *Hdh*Q150 homozygous (22 months) mice (Fig 2A and 2B and S2A and S2B Fig), suggesting that SIRT1 activity becomes impaired in the HD mouse brain. SIRT1 activity was also found to be compromised in the livers of 14 week old R6/2 and 22 month *Hdh*Q150 mice (S2C, S2D, S2E and S2F Fig).

SIRT1 does not co-localize with mutant HTT inclusions and is aberrantly phosphorylated in HD mice

Previous studies have shown that SIRT1 interacts with HTT *in vitro* [26]. To investigate whether the altered SIRT1 activity is caused by the sequestration of SIRT1 into HTT inclusions, we performed a double staining for SIRT1 and HTT (EM48) on nuclei isolated from the brains of 14-week R6/2 and 22-month *Hdh*Q150 homozygous mice, together with their age-matched WT littermates. Interestingly, SIRT1 did not co-localize with HTT inclusions (Fig 3A). To

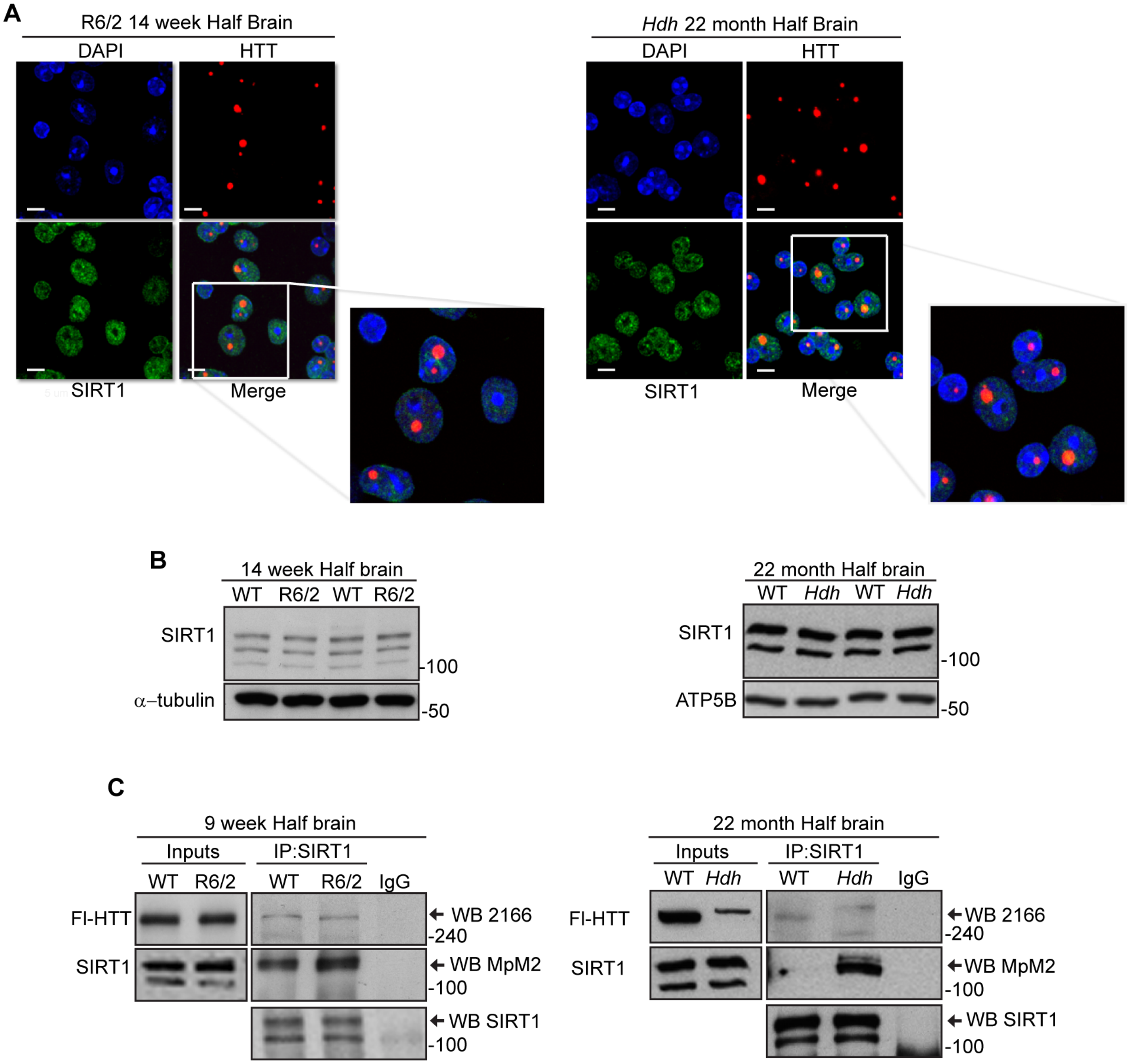

Fig 2. SIRT1 activity becomes reduced with disease progression in HD mouse models. (A) Representative immunofluorescence image of isolated nuclei extracted from half brains from R6/2 mice at 4 and 9 weeks of age and *Hdh*Q150 homozygotes at 2 and 22 months immunostained for SIRT1, P53 and AcP53 and counterstained with DAPI. (B) Relative intensity level of SIRT1, P53 and AcP53 from the immunostained nuclei shown in A. The quantification indicates that the level of acetylated P53 is higher in the HD models, consistent with a decrease in SIRT1 activity. Data are the mean ± SEM. ****p*<0.001. Scale bar, 10 μm; *Hdh* = *Hdh*Q150 homozygotes. n = 4 / genotype.

doi:10.1371/journal.pone.0145425.g002

further support this finding, the levels of SIRT1 protein were not decreased in HD brains as judged by western blot (Fig 3B).

The role of post-translational modifications (PTMs) in the regulation of SIRT1 activity has been the subject of several studies and phosphorylation has been described as a major control mechanism [41]. Therefore, to understand how mutant HTT reduces SIRT1 activity, we monitored the phosphorylation status of SIRT1 in HD mice. We performed SIRT1 immunoprecipitation from the brains of R6/2 mice at 9 weeks of age and *Hdh*Q150 homozygous mice at 22 months and probed the phosphorylation level of SIRT1 by western blot using the mitotic phosphoprotein monoclonal 2 (MpM2) antibody [42]. This antibody detects the phosphorylation

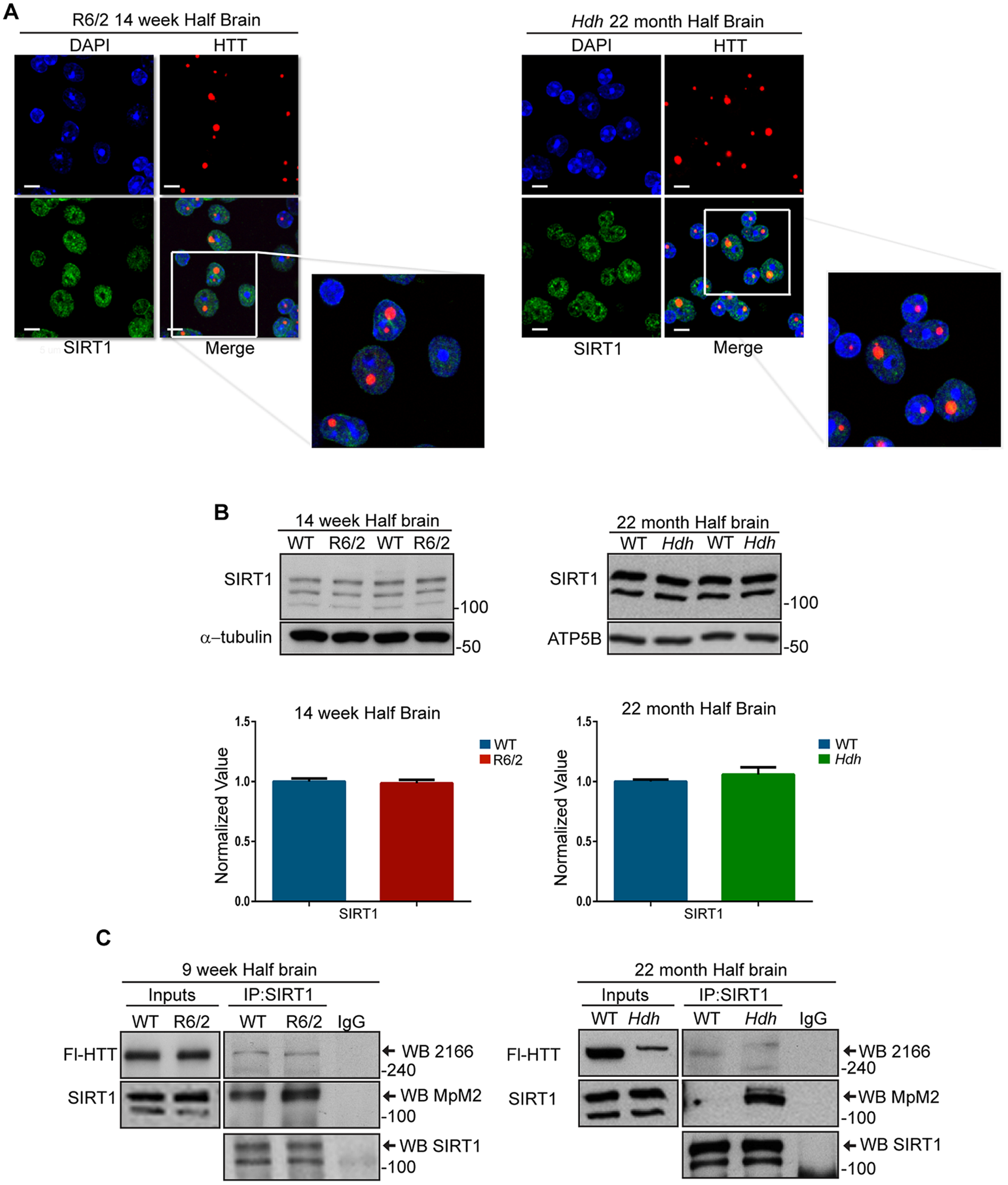

Fig 3. SIRT1 does not co-localize with HTT inclusions and is aberrantly phosphorylated in HD mice. (A) Representative immunofluorescence image of isolated nuclei extracted from half brains from R6/2 mice at 14 weeks of age and *Hdh*Q150 homozygotes at 22 months immunostained for both SIRT1 and mutant HTT (EM48), and counterstained with DAPI. (B) Representative western blot showing SIRT1 levels in half brains from R6/2 mice at 14 weeks of age and *Hdh*Q150 homozygotes at 22 months as compared to their WT littermates and relative quantification. (C) Western blots of SIRT1 and HTT after SIRT1 immunoprecipitation from half brain lysates of R6/2 and *Hdh*Q150 homozygotes as compared to their WT littermates. IP = immunoprecipitation; WB = western blotting. *Hdh* = *Hdh*Q150 homozygotes. Scale bar, 10 μm.

doi:10.1371/journal.pone.0145425.g003

of serine and threonine residues when they are followed by a proline (S/T-P sites) and it is not specific for a SIRT1 phosphorylation site (S3 Fig). Interestingly, a higher level of phosphorylated SIRT1 was found in the brains of both R6/2 and *Hdh*Q150 homozygotes as compared to their WT littermates (Fig 3C). As previously shown *in vitro* [26], we were able to co-immunoprecipitate endogenous HTT from R6/2 lysates and mutant HTT and WT HTT from *Hdh*Q150 homozygous and WT lysates respectively (Fig 3C). These results suggest that the impairment in SIRT1 function in the brains of HD mice is not related to its sequestration into HTT inclusions, but rather to an alteration in its phosphorylation profile.

SIRT1 phosphorylation becomes decreased in the striatum and increased in the cerebellum of HD mice

The analysis of total brain samples might mask or dilute any regional pathological changes. Therefore, we extended the analysis of SIRT1 phosphorylation to the striatum, cortex and cerebellum of R6/2 mice at 4, 9, and 14 weeks of age. We did not detect any difference in the phosphorylation status of SIRT1 at presymptomatic stages of the disease (i.e. 4-week-old R6/2) as compared to WT littermates in any brain region (Fig 4A and 4C and S4 Fig). In keeping with our functional data from total brain, the levels of phosphorylated SIRT1 were altered in the striatum and cerebellum of R6/2 mice by 9 weeks of age (Fig 4A and 4C). Surprisingly, the level of phosphorylation of SIRT1 remained unchanged in the R6/2 cortex at these later stages (S4 Fig), but notably was decreased in the striatum (Fig 4A) and increased in the cerebellum (Fig 4C) as compared to WT littermates. These data were replicated in the *Hdh*Q150 homozygous mice: there was no difference in the SIRT1 phosphorylation level at 2 months of age (Fig 4B and 4D) whereas SIRT1 phosphorylation was decreased in the striatum and increased in the cerebellum of 22-month-old *Hdh*Q150 homozygous mice (Fig 4B and 4D). Taken together, these results demonstrate that the presence of mutant HTT alters the phosphorylation status of SIRT1 in opposing directions for the striatum and cerebellum as the disease progresses.

Induction of SIRT1 activity is blocked in the striatum

Phosphorylation plays a central role in controlling protein activity, cellular localization and degradation [43]. To determine whether the differentially altered phosphorylation profile of SIRT1 in striatum and cerebellum corresponded to a compromised SIRT1 function in these brain regions, we immunostained for SIRT1, P53 and AcP53 in nuclei from the striatum and cerebellum of R6/2 and WT mice at 4, 9 and 14 weeks of age. Consistent with the total brain data, we did not detect a change in the intensity level of SIRT1 and P53 staining in either the striatum or cerebellum of R6/2 and WT mice, at any of the ages studied (S5A and S5B, S6A and S6B Figs). Interestingly, we observed a significant reduction in the level of AcP53 in the striatum of WT mice, corresponding to an increase in SIRT1 activity, between 4 and 9 weeks of age, which was absent in the striatum of R6/2 mice (Fig 5A and 5B). In contrast, when we analysed SIRT1 activity in the cerebellum, we detected no change in the level of AcP53 in WT samples at these ages and there was a significant increase in AcP53 in the cerebellum of R6/2 mice from 4 to 14 weeks of age (Fig 5C and 5D), corresponding to an impairment in SIRT1 activity. These data highlight that SIRT1 activity is regulated by different mechanisms in the striatum and cerebellum of WT mice between 4 and 14 weeks of age; SIRT1 activity is induced in the striatum between 4 and 9 weeks, whereas it remains constant in the cerebellum. The presence of mutant HTT can block this induction process in the striatum and cause a reduction in normal SIRT1 function in the cerebellum resulting in an impairment of SIRT1 activity in both brain regions (Fig 5).

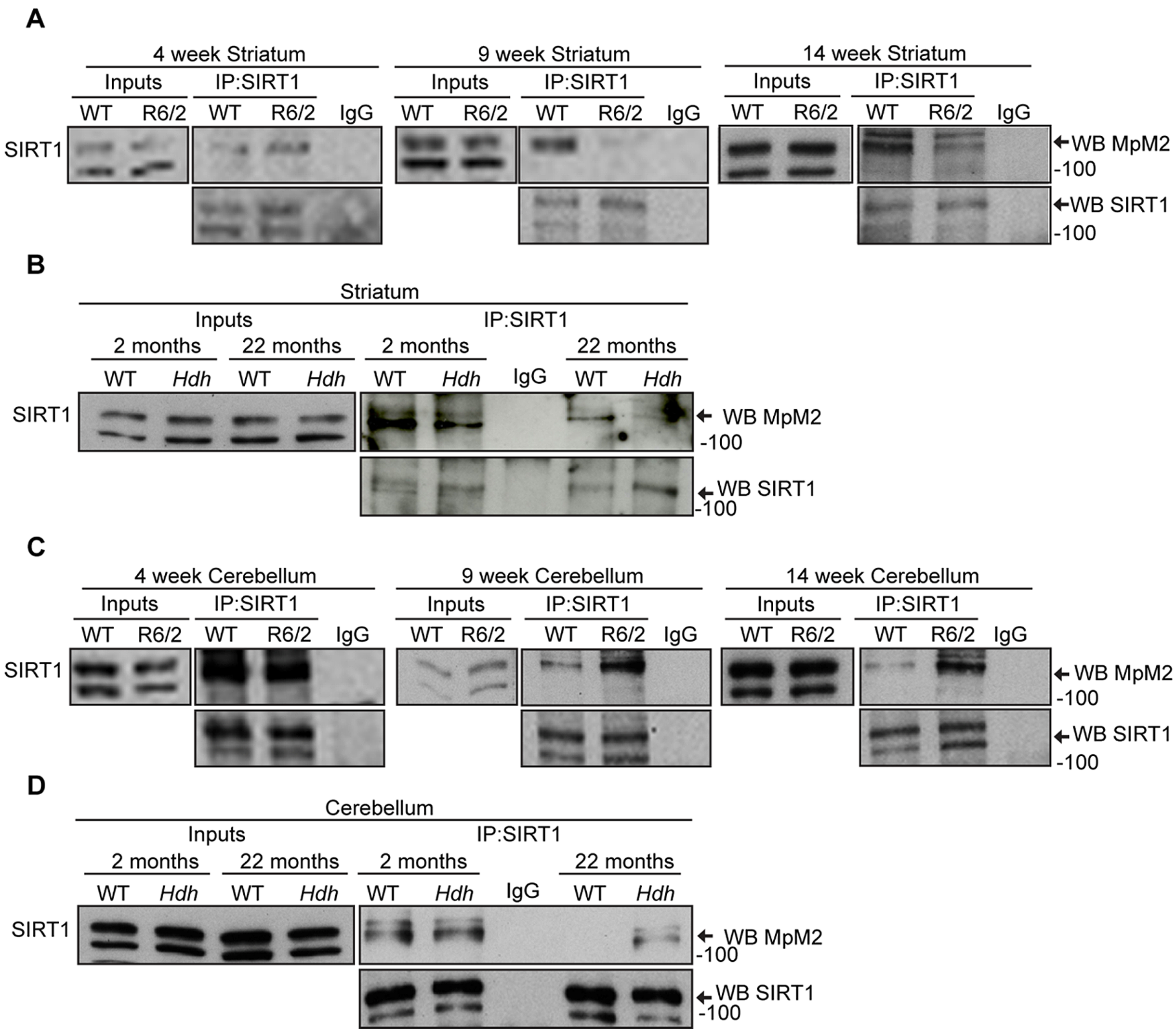

Fig 4. SIRT1 phosphorylation is altered in HD mouse models. (A, B) Western blots of SIRT1 and phosphorylated SIRT1 (MpM2) after SIRT1 immunoprecipitation from the striatum of R6/2 mice at 4, 9 and 14 weeks of age and *Hdh*Q150 homozygotes at 2 and 22 months as compared to their WT littermates. (C, D) Western blots of SIRT1 and phosphorylated SIRT1 (MpM2) after SIRT1 immunoprecipitation from the cerebellum of R6/2 mice at 4, 9 and 14 weeks of age and *Hdh*Q150 homozygotes at 2 and 22 months as compared to their WT littermates. IP = immunoprecipitation, WB = western blotting. *Hdh* = *Hdh*Q150 homozygotes. Data are representative of at least three independent experiments.

doi:10.1371/journal.pone.0145425.g004

SIRT1 induction in the striatum correlates with age-dependent phosphorylation

The comparison of SIRT1 activity in the striatum and cerebellum revealed that SIRT1 function is controlled by different mechanisms in these two brain regions in WT mice. In the striatum, SIRT1 is activated with age, a process that does not occur in the cerebellum. To monitor changes in the phosphorylation status of SIRT1 under normal physiological conditions we immunoprecipitated SIRT1 from striatal and cerebellar lysates of WT mice at 4, 9 and 14 weeks and immunoprobed with the MpM2 antibody. Notably, SIRT1 phosphorylation levels decreased in the striatum between 4 and 9 weeks of age (Fig 6A), a time at which the functional data revealed an increase in SIRT1 activity (Fig 5A and 5B). However, it then dramatically increased at 14 weeks (Fig 6A), a stage at which SIRT1 activity remains constant as compared to 9 weeks (Fig 5A and 5B). The

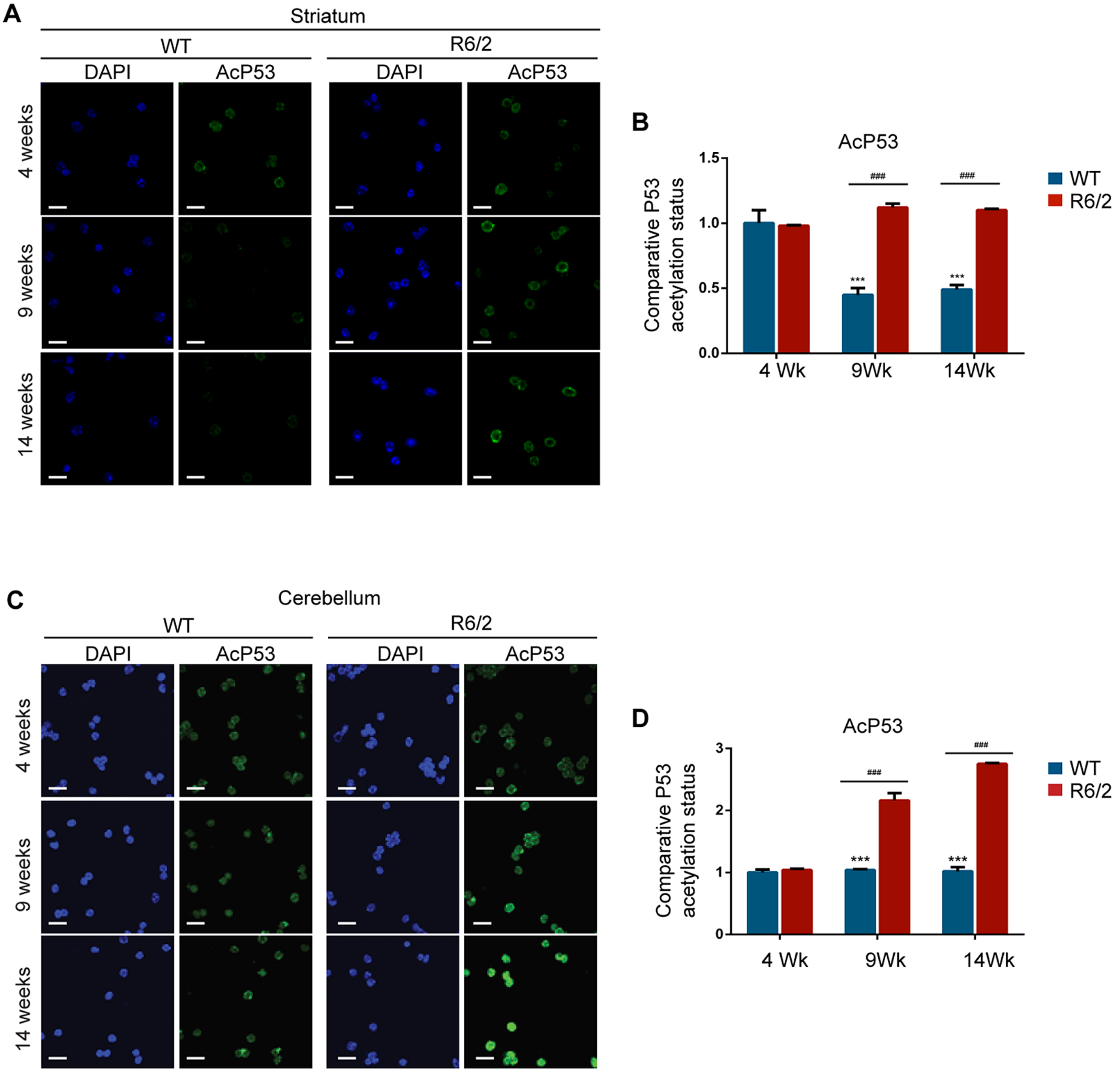

Fig 5. The induction of SIRT1 activity is blocked in the striatum in R6/2 mice. (A) Representative immunofluorescence images of isolated nuclei extracted from the striata of WT and R6/2 mice at 4, 9 and 14 weeks of age immunostained for AcP53 and counterstained with DAPI. (B) The relative fluorescent intensity level of AcP53 indicates that the level of acetylated P53 is lower in the striatum of WT mice as compared to the HD model, consistent with an increase in WT SIRT1 activity. (C) Representative immunofluorescence images of isolated nuclei extracted from the cerebella of WT and R6/2 mice at 4, 9 and 14 weeks of age immunostained for AcP53 and counterstained with DAPI. (D) The relative fluorescent intensity level of AcP53 indicates that the level of acetylated P53 is higher in the cerebellum of HD models, as compared to WT, consistent with a decrease in SIRT1 activity. Scale bar, 10 μm. Data are the mean ± SEM. ***P<0.001: statistically significant difference compared to 4 week WT. ###P<0.001: statistically significant difference between R6/2 and WT. n = 4 / genotype.

doi:10.1371/journal.pone.0145425.g005

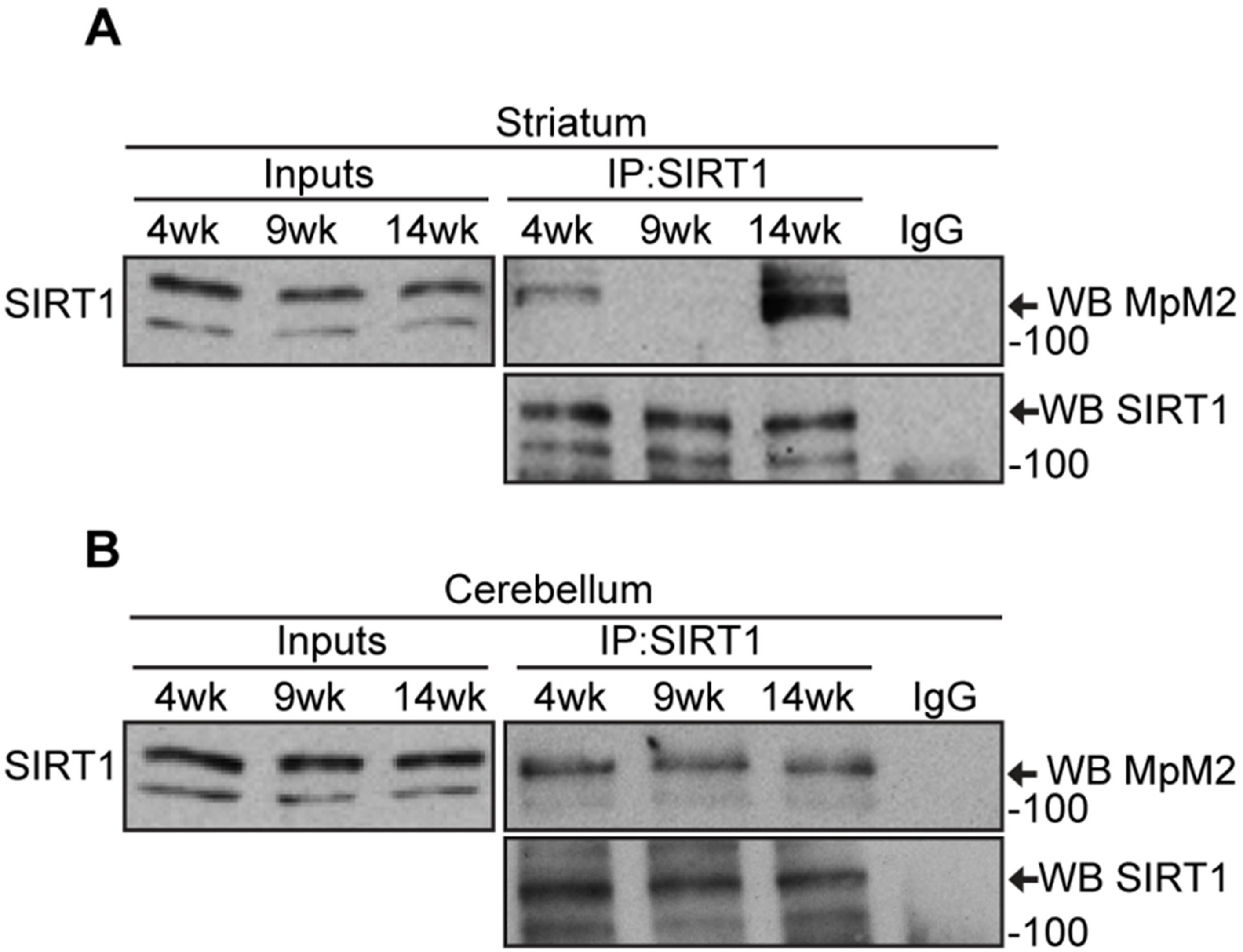

Fig 6. The striatum-specific regulatory mechanism of SIRT1 activity in HD mice is age-dependent. Western blots of SIRT1 and phosphorylated SIRT1 (MpM2) after SIRT1 immunoprecipitation from (A) the striatum of WT mice at 4, 9 and 14 weeks, and from (B) the cerebellum of WT mice at 4, 9 and 14 weeks; IP = immunoprecipitation; WB = western blot.

doi:10.1371/journal.pone.0145425.g006

MpM2 antibody detects phosphorylation on serine and threonine residues followed by proline (S/T-P sites) and is not specific for a SIRT1 phosphorylation site; therefore, the increased phosphorylation signal at 14 weeks may correspond to the phosphorylation of different SIRT1 residues to those detected a 4 and 9 weeks of age. Conversely, SIRT1 activity remains constant during these ages in the cerebellum (Fig 5C and 5D) and this is reflected by a phosphorylation level that does not change (Fig 6B). Taken together these data provide a link between the phosphorylation status of SIRT1 and its function, suggesting that in the striatum changes in the SIRT1 phosphorylation with age might be related to the induction of SIRT1 activity.

The sub-cellular distribution of SIRT1 is not altered in R6/2 mice

Previous studies suggested that the phosphorylation of human SIRT1 can increase its nuclear localization and enzymatic activity [44]. To assess whether the mis-regulation of SIRT1

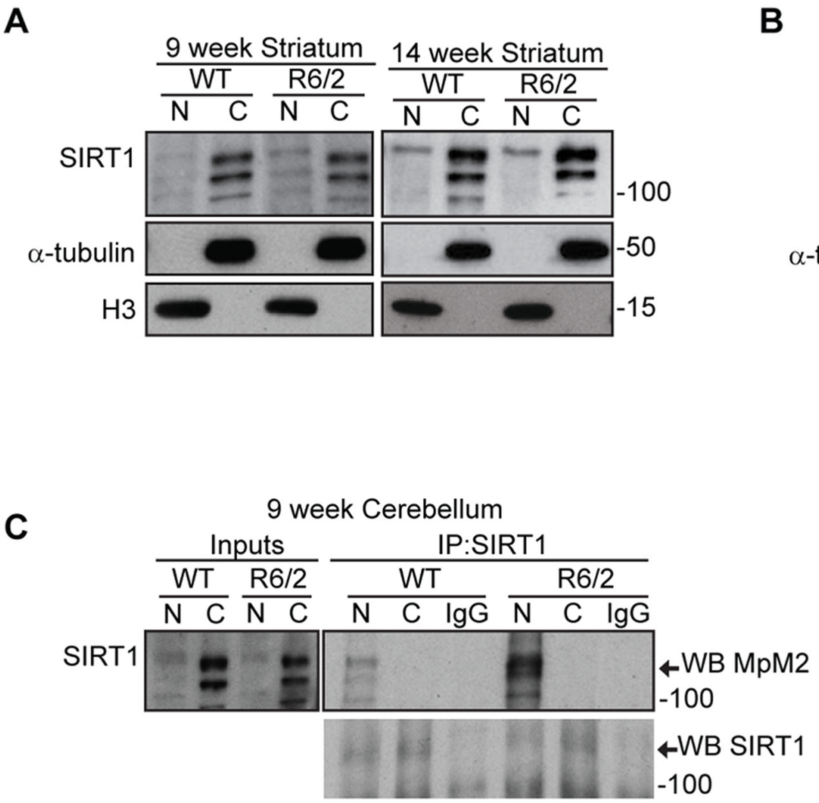

Fig 7. The sub-cellular distribution of SIRT1 is not altered in R6/2 mice. (A) Western blot for nuclear (N) and cytoplasmic (C) extracts from the striatum of R6/2 and WT mice at 9 weeks and 14 weeks of age immunoprobed with SIRT1, α-tubulin and histone H3. (B) Western blot for nuclear (N) and cytoplasmic (C) extracts from the cerebellum of R6/2 and WT mice at 9 weeks and 14 weeks of age immunoprobed with SIRT1, α-tubulin and histone H3. (C) Western blot of SIRT1 and phosphorylated SIRT1 (MpM2) after SIRT1 immunoprecipitation from nuclear and cytoplasmic fractions extracted from the cerebellum of R6/2 and WT mice at 9 weeks of age. IP = immunoprecipitation; WB = western blot.

doi:10.1371/journal.pone.0145425.g007

phosphorylation could affect its nuclear localization we prepared nuclear and cytoplasmic fractions from the striatum and cerebellum of R6/2 and WT mice at 9 and 14 weeks of age. Notably, we did not detect any difference in the distribution of SIRT1 at these ages between R6/2 and WT mice in either brain region (Fig 7A and 7B). However, the level of SIRT1 in the nuclear fraction was more pronounced at 14 weeks as compared to 9 weeks of age in the striatum and cerebellum of both R6/2 and WT mice (Fig 7A and 7B). We went on to analyse the phosphorylation level of SIRT1 in these two cellular compartments from the cerebellum by immunoprecipitation. This was not possible from the striatum due to limiting quantities of the extracts. Interestingly, a strong phosphorylation signal was detected in the nuclear fraction, that was absent from the cytoplasm, for both R6/2 and WT samples and, as previously shown on total lysates, the level of phosphorylation was much higher in R6/2 as compared to WT mice (Fig 7C). These results demonstrate that the sub-cellular distribution of SIRT1 is not affected by the presence of mutant HTT and suggests, once again, that the phosphorylation levels might be directly linked to the regulation of SIRT1 activity.

Tissue specific alteration of the subcellular distribution of AMPK-α1 with disease progression

Previous studies showed that DBC1 directly interacts with the catalytic domain of SIRT1 inhibiting its activity both *in vitro* and *in vivo* [19]. This dynamic interaction is sensitive to the

energetic state of the cell [19]. Activation of AMP-activated protein kinase-α1 (AMPK-α1), an important energy sensor in circumstances of low cellular energy, was recently shown to induce the activation of SIRT1 through the dissociation of SIRT1 and DBC1 [20,45]. To identify the possible role of AMPK-α1 and DBC1 in the molecular phenotypes described so far, we decided to study the interaction between these two opposing modulators of SIRT1 using co-immunoprecipitation. We immunoprecipitated AMPK-α1 from the striatum and cerebellum of 9-week R6/2, 22-month *Hdh*Q150 homozygous and WT littermates and detected the co-immunoprecipitated DBC1. Interestingly, we observed a stronger interaction between AMPK-α1 and DBC1 in the striatum of HD as compared to WT mice (Fig 8A), whereas equivalent amounts of DBC1 were co-immunoprecipitated with AMPK-α1 from cerebellar extracts of HD and WT samples (Fig 8B). These data suggest two possible scenarios: either the increased interaction of AMPK-α1 with the SIRT1-DBC1 complex might attempt to promote SIRT1 activation in the striatum of HD mice through the dissociation from DBC1, or the inability to induce SIRT1 in R6/2 mice might be due to an inhibitory retention of AMPK-α1 via DBC1.

To gain insight into the molecular events involved in this process we examined the cellular distribution of AMPK-α1 and DBC1 in the striatum and cerebellum of R6/2 and WT mice at 9 and 14 weeks of age. Consistent with the phenotypes described so far, the distributions AMPK-α1 and DBC1 were different in the striatum and cerebellum. Interestingly, using western blots of nuclear and cytoplasmic fractions, we were able to detect DBC1 in both cellular compartments and, although at 9 weeks of age DBC1 was slightly more abundant in the striatal cytoplasmic fraction, this balance was reverted by 14 weeks of age for both R6/2 and WT mice (Fig 8C). In contrast cerebellar DBC1 remained constant between the two cellular compartments at 9 and 14 weeks of age for both R6/2 and WT mice (Fig 8D). We next monitored the distribution of AMPK-α1 by immunostaining nuclei isolated from the striata of R6/2 and WT mice at 4, 9 and 14 weeks of age. At 9 weeks, AMPK-α1 was present in the nuclei from the WT striatum, whereas it could not be detected in nuclei from the striatum of R6/2 mice until 14 weeks of age (Fig 8E). Conversely, cerebellar extracts showed an early nuclear accumulation of AMPK-α1 in R6/2 at 9 weeks of age as compared to WT mice, where AMPK-α1 could only be detected in the nucleus at 14 weeks of age (Fig 8F). These data were confirmed by western blot (Fig 8C and 8D). The nuclear accumulation of AMPK-α1 at 9 weeks of age in the striatum of WT mice, in conjunction with an induction of SIRT1 activity, might indeed support a role for this kinase in the activation of SIRT1. This mechanism appears to be compromised in R6/2 mice and in this case, AMPK-α1 does not reach the nucleus until 14 weeks. Therefore, the increased interaction between AMPK-α1 and DBC1 might result in the retention of AMPK-α1 in the cytoplasm inhibiting the activation of SIRT1, and/or attempting to rescue SIRT1 activity by preventing DBC1 from binding to SIRT1. Conversely, the early nuclear accumulation of AMPK-α1 in the cerebellum of R6/2 at 9 weeks of age as compared to WT mice with the concomitant alteration in SIRT1 function might be an attempt to increase impaired SIRT1 activity. Taken together these data suggest that the inhibition of SIRT1 function in the striatum of R6/2 might arise through an altered functionality of AMPK-α1 and that AMPK-α1 might be involved in rescuing a deficient SIRT1 function both in the striatum and cerebellum, although through different molecular mechanisms.

SIRT1, AMPK-α1 and DBC1 act as partners in the same regulatory circuit to control SIRT1 activity in the striatum

Our data suggest that AMPK-α1 may play an active role in attempting to rescue SIRT1 deficiency in both the striatum and cerebellum of R6/2 mice. To obtain further evidence for a regulatory circuit involving these three proteins, we went on to compare the expression levels of

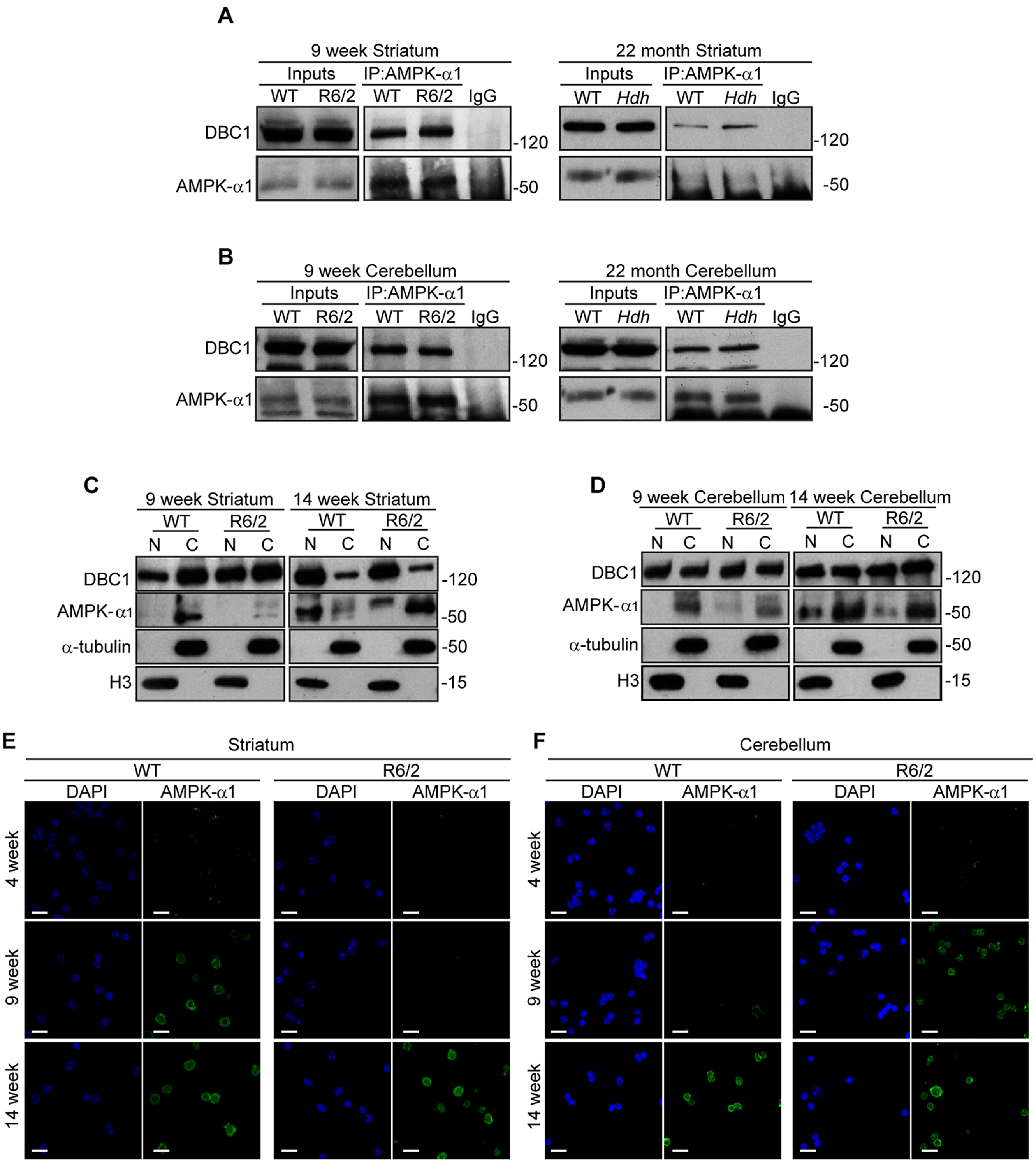

Fig 8. The DBC1-AMPK-α1 interaction is increased in the striatum of HD mice. (A-B) Western blots of DBC1 and AMPK-α1 after AMPK-α1 immunoprecipitation from (A) the striatum of 9-week R6/2 and 22-month *Hdh*Q150 homozygotes, and (B) the cerebellum of 9-week R6/2 and 22-month *Hdh*Q150 homozygotes. (C-D) Western blot for DBC1, AMPK-α1, α-tubulin and histone H3 of nuclear (N) and cytoplasmic (C) extracts from (C) striatum and (D) cerebellum of WT and R6/2 mice at 9 and 14 weeks of age. (E-F) Representative immunofluorescence image of nuclei extracted from (E) striatum and (F) cerebellum of WT and R6/2 mice at 4, 9 and 14 weeks of age immunostained for AMPK-α1 and counterstained with DAPI. Scale bar, 10 μm.
IP = immunoprecipitation.

doi:10.1371/journal.pone.0145425.g008

SIRT1, DBC1 and AMPK-α1 in the striatum and cerebellum of R6/2 at 4, 9 and 14 weeks of age and *Hdh*Q150 homozygotes at 2 and 22 months as compared to their WT littermates. Strikingly, there was a synchronised, statistically significant down-regulation (35–40%) of all three genes at the mRNA level from 4 to 9 weeks of age in the striatum of WT mice (Fig 9A). We detected the same significant reduction in the striatum of R6/2 mice for *Dbc1* and *Ampk*-α1, and there was a weak trend for *Sirt1* (Fig 9A). Notably, the presence of this regulatory circuit in the cerebellum was not supported by the same co-ordinated changes in expression levels, although the expression level of *Sirt1* was significantly higher in WT mice at 9 and 14 weeks as compared to 4 weeks of age (Fig 9D).

These mRNA changes did not result in concomitant alterations in the levels of the SIRT1, DBC1, and AMPK-α1 proteins (Fig 9B, 9C, 9E and 9F). The mRNA changes in the striatum may be the result of a stabilisation of these proteins between 4 and 9 weeks of age. Indeed, the levels of all three proteins were equivalent at 2 and 22 months in the *Hdh*Q150 homozygotes and WT mice (S7A and S7B Fig). Interestingly, we observed a significant upregulation of AMPK-α1 in the striatum of WT mice between 4 and 9 weeks occurring in conjunction with the increase in SIRT1 activity, neither of which took place in R6/2 mice (Fig 9B and 9C). The only change that occurred in the cerebellum was a reduction in the level of SIRT1 in both WT and R6/2 at 14 weeks of age (Fig 9E and 9F).

Dbc1 ablation does not improve HD-related phenotypes

Our data indicate that brain region specific dysregulated cellular processes result in a reduction in SIRT1 activity in the brains of HD mouse models. We hypothesis that this is related to the phosphorylation status of SIRT1 and that the AMPK-α1 kinase attempts to rescue SIRT1 function. As DBC1 is a negative regulator of SIRT1, and *Dbc1* knock-out mice are viable and healthy [19], we elected to use a genetic approach to ablate DBC1 levels in R6/2 mice and investigate whether the dissociation between SIRT1 and DBC1 could increase SIRT1 activity and improve HD phenotypes. We crossed R6/2 transgenic mice with *Dbc1* heterozygous knock-out mice ($Dbc1^{+/-}$) to obtain $Dbc1^{+/-}$::R6/2 males that were then crossed with $Dbc1^{+/-}$ females to generate WT, $Dbc1^{+/-}$, $Dbc1^{-/-}$, R6/2, $Dbc1^{+/-}$::R6/2 and $Dbc1^{-/-}$::R6/2 mice. As predicted, genetic ablation of *Dbc1* resulted in a significant decrease in *Dbc1* mRNA and DBC1 protein levels, and we found that this did not alter the expression of SIRT1 (S8A Fig). To confirm that the removal of DBC1 resulted in an increase in SIRT1 activity, we immunostained nuclei extracted from the brains of WT, $Dbc1^{-/-}$, R6/2 and $Dbc1^{-/-}$::R6/2 mice at 9 weeks of age for SIRT1, P53, AcP53 and DBC1 and counterstained with DAPI. The absence of DBC1 did not affect the level and nuclear accumulation of SIRT1 and/or P53 (Fig 10A and 10B). As expected, the ablation of DBC1 in WT mice resulted in an increase in SIRT1 activity as indicated by a significant reduction (~65%) in the signal intensity for AcP53 in $Dbc1^{-/-}$ as compared to WT mice (Fig 10B). SIRT1 activity was decreased in R6/2 mice (consistent with Fig 2) and surprisingly the absence of DBC1 did not ameliorate this impairment (Fig 10). In line with these results, we did not detect improvements in the onset and progression of specific behavioural HD-related phenotypes such as body weight, grip strength and rotarod impairment (S8B, S8C and S8D Fig). Taken together these results suggest that the negative effect of mutant HTT on SIRT1 activity might be multifactorial and/or operate outside the inhibitory circuit controlled by DBC1.

Discussion

The involvement of SIRT1 in lifespan extension and cellular protection from aggregation-prone proteins http://jcb.rupress.org/content/190/5/719.full-ref-91 has made it a promising

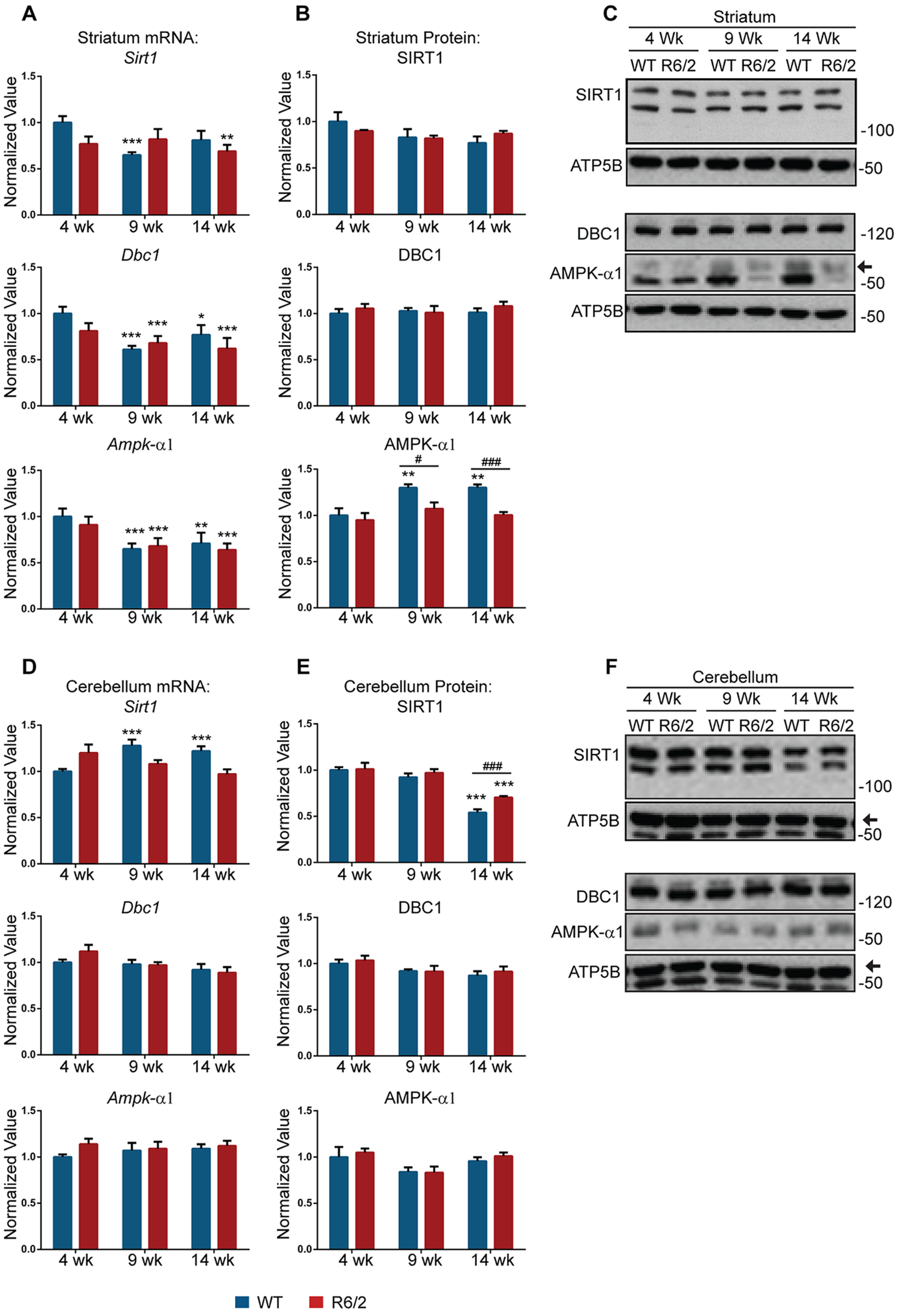

Fig 9. Expression analysis of SIRT1, DBC1 and AMPK-α1 in R6/2 mice. qPCR analysis of the expression level of *Sirt1*, *Dbc1* and *Ampk-α1* in (A) striatum and (D) cerebellum of R6/2 and WT mice at 4, 9 and 14 weeks of age. Values were calculated relative to 4-week WT mice. Relative protein level of SIRT1, DBC1 and AMPK-α1 in (B) striatum and (E) cerebellum of R6/2 and WT mice at 4, 9 and 14 weeks of age. Densitometric values were calculated relatively to 4-week WT mice. Representative western blots for SIRT1, AMPK-α1 and DBC1 in (C) striatum and (F) cerebellum of R6/2 and WT mice at 4, 9 and 14 weeks of age. Data are the mean ± SEM. *P<0.05; **P<0.01; ***P<0.001: statistically significant difference as compared to WT at 4 weeks of age. #P<0.05; ###P<0.001: statistically significant difference between WT and R6/2. For qPCR n = 8 / genotype; for western blot n = 4 / genotype.

doi:10.1371/journal.pone.0145425.g009

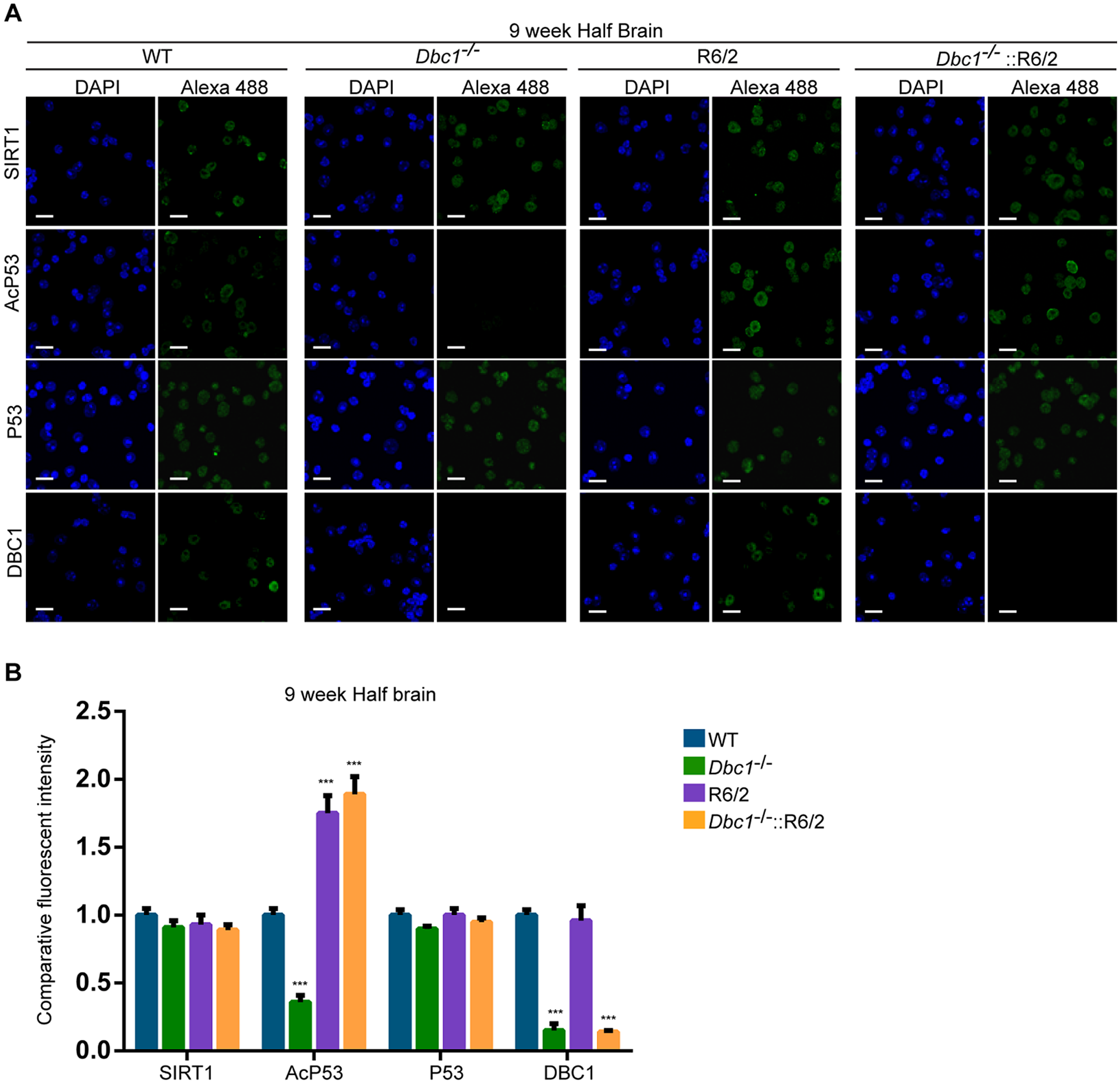

Fig 10. *Dbc1* ablation does not ameliorate the impairment in SIRT1 activity in the brains of R6/2 mice. (A) Representative immunofluorescence image of nuclei extracted from half brain of WT, *Dbc1*$^{-/-}$, R6/2 and *Dbc1*$^{-/-}$::R6/2 mice at 9 weeks of age immunostained for SIRT1, AcP53, P53 and DBC1 and counterstained with DAPI. (B) Relative intensity level of SIRT1, AcP53, P53 and DBC1 immunostaining in A. The quantification indicates that the level of acetylated P53 is lower in *Dbc1*$^{-/-}$ mice and higher in R6/2 mice as compared to WT, consistent with a respective increase and decrease in SIRT1 activity. Scale bar: 10 μm. Data are mean ± SEM. ***P<0.001 statistically significant as compared to WT. n = 4 / genotype.

doi:10.1371/journal.pone.0145425.g010

therapeutic target for neurodegenerative disorders [46–48]. In the context of HD, the manipulation of SIRT1 activity has not generated results that are easy to interpret. On the one hand, over expression of SIRT1 has been shown to reduce mutant HTT-induced toxicity in HD mouse models, improving motor function and reducing brain atrophy [25,26]. In contrast to

this, the pharmacological inhibition of SIRT1 has been shown to have beneficial effects in *drosophila* and mouse models of HD [29] and on the basis of these results, selisistat was assessed for safety and tolerability in a clinical trial aimed at the development of HD pharmacodynamic biomarkers [30]. Despite this interest, the integrity of SIRT1 function in HD has not been comprehensively investigated. In the present study, we have shown that SIRT1 activity is impaired in different brain regions from two distinct mouse models of HD and that this is linked to an altered SIRT1 phosphorylation status. Furthermore, we provide insights into the temporal tissue-specific regulation of SIRT1 activity in different brain regions from WT mice.

To monitor SIRT1 activity in the brain, we analysed P53 acetylation by performing immunohistochemistry on nuclei isolated from both R6/2 and *Hdh*Q150 mice as compared to their WT littermates. We did not detect an alteration in SIRT1 function at the presymptomatic stage in either model. However, SIRT1 activity was overtly compromised by 9 weeks of age in the R6/2 mice with a comparable impairment at late stage disease in both models. This was not caused by SIRT1 sequestration into HTT inclusions and we did not detect any variation in either the level or sub-cellular distribution of SIRT1 between HD and WT mice. We also showed that this impairment occurred in liver and therefore extends to peripheral tissues. We would not expect the increase in P53 acetylation to be caused by the HD-related dysregulation of acetyltransferases as it has been shown that the P53 acetyltransferases: CREB binding protein, P300 and P300/CBP associated factor, are inhibited with disease progression in HD [49] and would therefore be expected to result in a reduction in P53 acetylation, the opposite to that observed in this study. We were unable to replicate these results using an independent measure of SIRT1 activity as the commercial kit that we tested was not specific for SIRT1 in mouse brain lysates.

The role of post-translational modifications in the regulation of SIRT1 activity has been the subject of several studies and phosphorylation has been described as a major control mechanism [41]. It has been shown that kinases such as JNK1 and CK2 can phosphorylate SIRT1 thereby increasing its nuclear deacetylase activity [44,50]. The phosphorylation of SIRT1 by JNK1 has also been shown to induce SIRT1 ubiquitination and proteasomal degradation [44]. In this study, although we detected an impaired SIRT1 activity in both the striatum and cerebellum of HD mice, the phosphorylation level of SIRT1 changed in opposite directions in these two brain regions, indicative of a tissue-specific SIRT1 regulation. Our further investigations led us to identify a striatum-specific phosphorylation-dependent induction of SIRT1 activity with age in WT mice, which does not occur in the cerebellum. Taken together, our findings suggest that it is the induction of SIRT1 function that is compromised by mutant HTT in the striatum of HD mice (Fig 11A), whereas in the cerebellum, mutant HTT impairs an already established SIRT1 activity.

In an attempt to rescue this SIRT1 deficiency, we crossed R6/2 mice with *Dbc1*$^{-/-}$ mice, as DBC1 negatively regulates SIRT1 via the direct interaction with its deacetylase domain [19]. Strikingly, despite a significant upregulation of SIRT1 activity in *Dbc1*$^{-/-}$ mice, the ablation of DBC1 from R6/2 mice had no effect on the impairment of SIRT1 activity, suggesting that mutant HTT alters key regulatory events that lie outside the inhibitory circuit controlled by DBC1. Consistent with this, the absence of DBC1 did not lead to improvements in the onset and progression of several behavioural HD-related phenotypes.

In contrast to DBC1, AMPK-α1 has been reported to positively regulate the activity of SIRT1 by inducing SIRT1 activation through its dissociation from DBC1 [20,45]. In addition, there is evidence to indicate that AMPK-α1 and SIRT1 can regulate each other [21]. Interestingly, our co-immunoprecipitation experiments revealed an increased interaction between DBC1 and AMPK-α1 in the striatum of HD mice, which might point to an attempt to rescue SIRT1 function. On the other hand, the nuclear accumulation of AMPK-α1 is delayed in the striatal nuclei of R6/2 mice and its retention in the cytoplasm through an interaction with DBC1 might impede

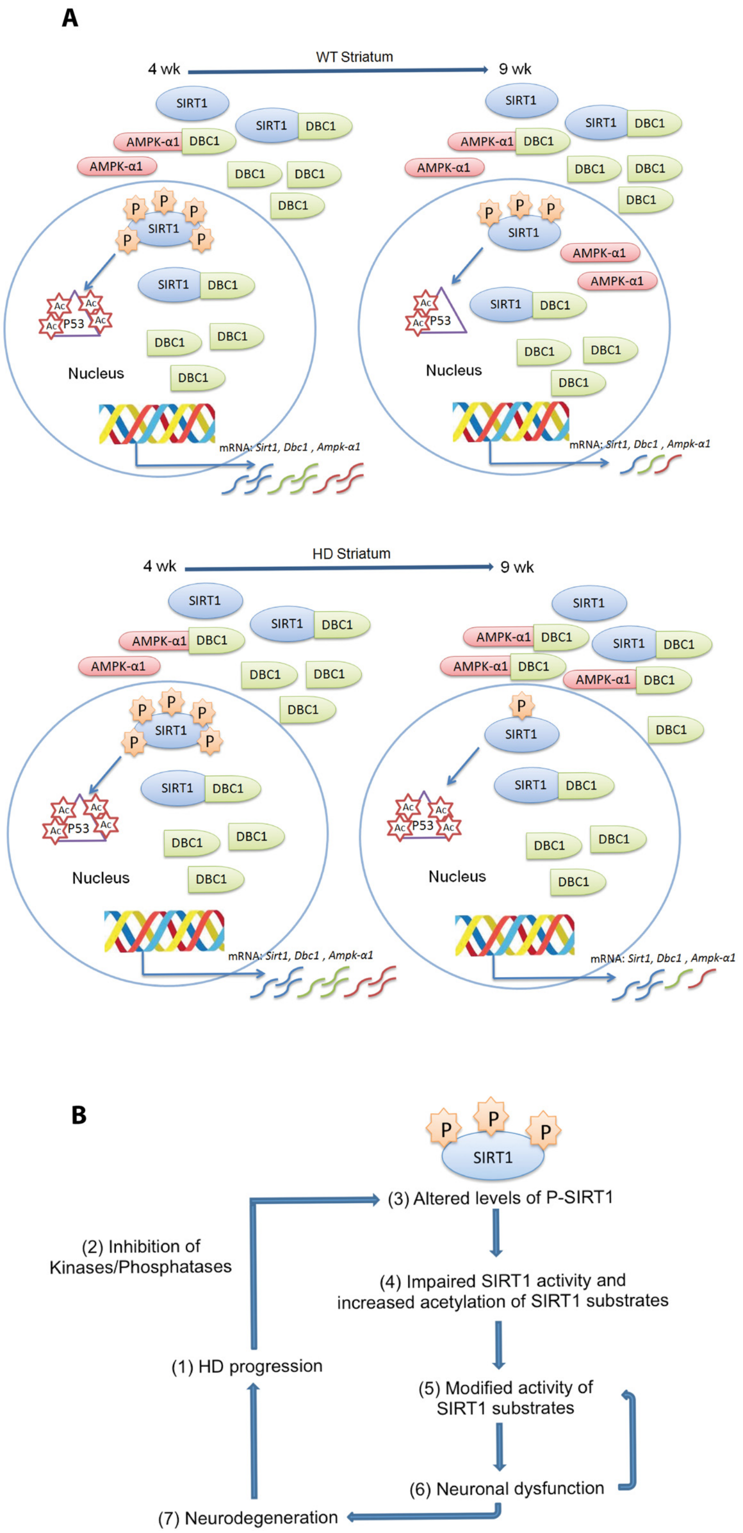

Fig 11. Proposed model for the striatum-specific regulation of SIRT1 via phosphorylation in WT mice and for the impairment in SIRT1 activity in HD brain. (A) The change in SIRT1 phosphorylation status in the striatum of WT mice between 4 and 9 weeks of age induces an increase in SIRT1 activity followed by a reduction in acetylated P53. The nuclear accumulation of AMPK-α1 in WT striatum at 9 weeks supports a role for this kinase in the activation of SIRT1 (AMPK-α1 is not the kinase involved in the change in SIRT1

phosphorylation detected here, as the MpM2 antibody only recognises Ser/Thr-Pro residues and AMPK-α1 does not phosphorylate Ser/Thr residues that are followed by proline). AMPK-α1 is present in the nucleus at 9 weeks and activates SIRT1 through a mechanism independent of DBC1. The down-regulation of *Sirt1*, *Dbc1* and *Ampk-α1* at the mRNA level between 4 and 9 weeks of age is consistent with these three proteins being partners in the same regulatory circuit. In the context of HD, the marked reduction in SIRT1 phosphorylation impedes the induction of SIRT1 activity. The greater interaction between AMPK-α1 and DBC1 may result in the cytoplasmic retention of AMPK-α1, inhibiting the activation of SIRT1, and/or promoting a futile rescue attempt by preventing DBC1 from binding to SIRT1. (B) The HD pathogenic process leads to an alteration in the phosphorylation status of SIRT1, resulting in an impairment in SIRT1 activity which modulated the function of SIRT1 targets that include P53 and may contribute to neuronal dysfunction.

doi:10.1371/journal.pone.0145425.g011

SIRT1 activation. In contrast, the nuclear accumulation of AMPK-α1 in the cerebellum of R6/2 mice occurs earlier than in WT mice, indicating that it might be attempting to relieve the SIRT1 inhibition imposed by mutant HTT. In support of the existence of a striatum-specific regulatory circuit linking these three proteins in the induction of SIRT1 activity, we found that the downregulation of *Sirt1*, *Dbc1* and *Ampk*-α1 is co-ordinated in WT mice between 4 and 9 weeks of age. The protein level of AMPK-α1 increases in WT mice during the same time frame, which correlates with the induction of SIRT1 activity, neither of which occurs in R6/2 mice.

We propose a model whereby disease progression leads to an altered SIRT1 phosphorylation status. As a consequence, the decrease in SIRT1 activity leads to a reduction in the deacetylation of P53 and other SIRT1 substrates, modifications that may contribute to neuronal dysfunction (Fig 11B). This would be consistent with previous data showing that the ablation of P53 from an HD mouse model had beneficial consequences [51]. In conclusion, our data provide two major new findings. First, we have shown that mechanisms controlling the tissue-specific regulation of SIRT1 activity differ between brain regions, and we have identified a novel striatum-specific phosphorylation-dependent mechanism of SIRT1 induction in WT mice. Second, we demonstrate that SIRT1 activity is impaired in two distinct HD mouse models. Given that SIRT1 plays a central role in metabolism, longevity and neurodegeneration, loss of SIRT1 activity may contribute significantly to disease progression in HD. These results provide new insights into the mechanisms that regulate SIRT1 function and may lead to the development of new strategies by which SIRT1 can be manipulated for therapeutic benefit.

Supporting Information

S1 ARRIVE Checklist. NC3Rs ARRIVE Guidelines checklist.
(PDF)

S1 Fig. Measurement of SIRT1 activity with the Fluor de Lys assay. Fluorescence signal obtained after 0, 20, 40 and 60 minute incubations at 37°C. 25 μg of protein from cortical lysates of WT or *Sirt1*KO mice and 2U/well of SIRT1-recombinant protein were incubated with (A) 50, (B) 100 or (C) 200 μM of substrate and with 200 μM of NAD^+ and 5 μM TSA. n = 2 / genotype. AFU = *arbitrary fluorescence units*.
(TIF)

S2 Fig. SIRT1 activity becomes reduced in HD mouse models. Representative immunofluorescence image of isolated nuclei extracted from (A) half brain and (C) liver from R6/2 mice at 14 weeks of age and (E) liver from 22 month *Hdh*Q150 mice immunostained for SIRT1, P53 and AcP53 and counterstained with DAPI. (B, D, F) Relative intensity levels of SIRT1, p53 and Acp53 from immunostained nuclei in (A, C, E) respectively. The quantification indicates that the level of acetylated P53 is higher in the HD models, consistent with a decrease in SIRT1 activity as depicted in Fig 2C. Scale bar, 10 μm. n = 4 / genotype.
(TIF)

S3 Fig. Mouse sequence of SIRT1 indicating residues detected by the MpM2 antibody. Ser-Pro residues are highlight in yellow and Thr-Pro residues are highlight in green. Notably, all the phosphorylation sites recognized by the Mpm2 antibody are located in the N-terminal (1-244aa, purple) and C-terminal (498-737aa, orange) regions. The catalytic domain of SIRT1 is underlined in blue.
(TIF)

S4 Fig. SIRT1 phosphorylation is altered in HD mouse models. Western blots of SIRT1 and phosphorylated SIRT1 (MpM2) after SIRT1 immunoprecipitation from cortical lysates of R6/2 mice at 4, 9 and 14 weeks of age compared to WT littermates. IP = immunoprecipitation, WB = western blotting.
(TIF)

S5 Fig. The induction of SIRT1 activity is blocked in the striatum. Representative immunofluorescence images of isolated nuclei extracted from the striata of WT and R6/2 mice at 4, 9 and 14 weeks of age immunostained for (A) SIRT1 and (B) P53, and counterstained with DAPI. The relative intensity levels of SIRT1 and P53 are depicted alongside. Scale bar, 10 μm. Data are the mean ± SEM. n = 10 / genotype in 2 pools of 5 striata.
(TIF)

S6 Fig. SIRT1 activity is impaired in the cerebellum. Representative immunofluorescence image of isolated nuclei extracted from the cerebella of WT and R6/2 mice at 4, 9 and 14 weeks of age immunostained for (A) SIRT1 and (B) P53, and counterstained with DAPI. The relative intensity levels of SIRT1 and P53 are depicted alongside. Scale bar, 10 μm. Data are the mean ± SEM. n = 10 / genotype in 2 pools of 5 cerebella.
(TIF)

S7 Fig. Analysis of the expression levels of SIRT1, DBC1 and AMPK-α1 in *Hdh*Q150 homozygous mice. (A) Relative protein levels and (B) representative western blots of SIRT1, DBC1 and AMPK-α1 in striatum and cerebellum of 2 and 22-month *Hdh*Q150 homozygotes and WT mice. Densitometric values were calculated relative to 2-month WT mice. Data are mean ± SEM. *Hdh* = *Hdh*Q150 homozygotes. n = 4 / genotype.
(TIF)

S8 Fig. *Dbc1* ablation does not delay the onset and progression of HD phenotypes. (A) Analysis of DBC1 and SIRT1 expression in 4-week cortex of WT, *Dbc1*$^{+/-}$ and *Dbc1*$^{-/-}$ mice. (B) Body weight, (C) forelimb grip strength and (D) latency to fall from the rotarod were measured from 4 to 14 weeks of age. Number of mice: WT: 22 (7M-15F), *Dbc1*$^{+/-}$: 19 (11M-8F), *Dbc1*$^{-/-}$: 18 (9M-9F), R6/2: 22 (13M-9F), *Dbc1*$^{+/-}$::R6/2: 18 (9M-9F), *Dbc1*$^{-/-}$::R6/2: 19 (6M-13F). Data are the mean ± SEM. $^{***}P<0.001$: statistically significant difference as compared to WT.
(TIF)

S1 Table. Summary of the working dilution and application of all antibodies.
(DOCX)

S2 Table. Summary of in-house designed primer and probe sequences for qPCR.
(DOCX)

Acknowledgments

We thank David Holzman for the SIRT1 transgenic mice and Ivan Rattray for help with statistical analysis.

Author Contributions

Conceived and designed the experiments: RT AC GPB. Performed the experiments: RT. Analyzed the data: RT AC GPB. Contributed reagents/materials/analysis tools: ACB NJ DLS ENC. Wrote the paper: RT AC GPB.

References

1. HDCRG (1993) A novel gene containing a trinucleotide repeat that is expanded and unstable on Huntington's disease chromosomes. The Huntington's Disease Collaborative Research Group [see comments]. Cell 72: 971–983. PMID: 8458085
2. Novak MJ, Tabrizi SJ (2010) Huntington's disease. BMJ 340: c3109. doi: 10.1136/bmj.c3109 PMID: 20591965
3. Waldvogel HJ, Kim EH, Tippett LJ, Vonsattel JP, Faull RL (2014) Neuropathology in the human brain. In: Bates GP, Tabrizi SJ, Jones L, editors. Huntington's Disease. 4th ed. New York: Oxford University Press. pp. 185–217.
4. Bates GP, Landles C (2014) Preclinical experimental therapeutics. In: Bates GP, Tabrizi SJ, Jones L, editors. Huntington's Disease. 4th ed. NewYork: Oxford University Press. pp. 410–461.
5. Mangiarini L, Sathasivam K, Seller M, Cozens B, Harper A, Hetherington C, et al. (1996) Exon 1 of the HD gene with an expanded CAG repeat is sufficient to cause a progressive neurological phenotype in transgenic mice. Cell 87: 493–506. PMID: 8898202
6. Sathasivam K, Neueder A, Gipson TA, Landles C, Benjamin AC, Bondulich MK, et al. (2013) Aberrant splicing of HTT generates the pathogenic exon 1 protein in Huntington disease. Proc Natl Acad Sci U S A 110: 2366–2370. doi: 10.1073/pnas.1221891110 PMID: 23341618
7. Lin CH, Tallaksen-Greene S, Chien WM, Cearley JA, Jackson WS, Crouse AB, et al. (2001) Neurological abnormalities in a knock-in mouse model of Huntington's disease. Hum Mol Genet 10: 137–144. PMID: 11152661
8. Landles C, Sathasivam K, Weiss A, Woodman B, Moffitt H, Finkbeiner S, et al. (2010) Proteolysis of mutant huntingtin produces an exon 1 fragment that accumulates as an aggregated protein in neuronal nuclei in Huntington disease. J Biol Chem 285: 8808–8823. doi: 10.1074/jbc.M109.075028 PMID: 20086007
9. Woodman B, Butler R, Landles C, Lupton MK, Tse J, Hockly E, et al. (2007) The Hdh(Q150/Q150) knock-in mouse model of HD and the R6/2 exon 1 model develop comparable and widespread molecular phenotypes. Brain Res Bull 72: 83–97. PMID: 17352931
10. Moffitt H, McPhail GD, Woodman B, Hobbs C, Bates GP (2009) Formation of polyglutamine inclusions in a wide range of non-CNS tissues in the HdhQ150 knock-in mouse model of Huntington's disease. PLoS One 4: e8025. doi: 10.1371/journal.pone.0008025 PMID: 19956633
11. Kuhn A, Goldstein DR, Hodges A, Strand AD, Sengstag T, Kooperberg C, et al. (2007) Mutant huntingtin's effects on striatal gene expression in mice recapitulate changes observed in human Huntington's disease brain and do not differ with mutant huntingtin length or wild-type huntingtin dosage. Hum Mol Genet 16: 1845–1861. PMID: 17519223
12. Labbadia J, Cunliffe H, Weiss A, Katsyuba E, Sathasivam K, Seredenina T, et al. (2011) Altered chromatin architecture underlies progressive impairment of the heat shock response in mouse models of Huntington disease. J Clin Invest 121: 3306–3319. doi: 10.1172/JCI57413 PMID: 21785217
13. Mielcarek M, Inuabasi L, Bondulich MK, Muller T, Osborne GF, Franklin SA, et al. (2014) Dysfunction of the CNS-heart axis in mouse models of Huntington's disease. PLoS Genet 10: e1004550. doi: 10.1371/journal.pgen.1004550 PMID: 25101683
14. Mielcarek M, Toczek M, Smeets CJ, Franklin SA, Bondulich MK, Jolinon N, et al. (2015) HDAC4-myogenin axis as an important marker of HD-related skeletal muscle atrophy. PLoS Genet 11: e1005021. doi: 10.1371/journal.pgen.1005021 PMID: 25748626
15. Cohen HY, Miller C, Bitterman KJ, Wall NR, Hekking B, Kessler B, et al. (2004) Calorie restriction promotes mammalian cell survival by inducing the SIRT1 deacetylase. Science 305: 390–392. PMID: 15205477
16. Cha YI, Kim HS (2013) Emerging role of sirtuins on tumorigenesis: possible link between aging and cancer. BMB Rep 46: 429–438. PMID: 24064057
17. Rodgers JT, Lerin C, Haas W, Gygi SP, Spiegelman BM, Puigserver P (2005) Nutrient control of glucose homeostasis through a complex of PGC-1alpha and SIRT1. Nature 434: 113–118. PMID: 15744310

18. Brooks CL, Gu W (2009) How does SIRT1 affect metabolism, senescence and cancer? Nat Rev Cancer 9: 123–128. doi: 10.1038/nrc2562 PMID: 19132007

19. Escande C, Chini CC, Nin V, Dykhouse KM, Novak CM, Levine J, et al. (2010) Deleted in breast cancer-1 regulates SIRT1 activity and contributes to high-fat diet-induced liver steatosis in mice. J Clin Invest 120: 545–558. doi: 10.1172/JCI39319 PMID: 20071779

20. Nin V, Escande C, Chini CC, Giri S, Camacho-Pereira J, Matalonga J, et al. (2012) Role of deleted in breast cancer 1 (DBC1) protein in SIRT1 deacetylase activation induced by protein kinase A and AMP-activated protein kinase. J Biol Chem 287: 23489–23501. doi: 10.1074/jbc.M112.365874 PMID: 22553202

21. Ruderman NB, Xu XJ, Nelson L, Cacicedo JM, Saha AK, Lan F, et al. (2010) AMPK and SIRT1: a long-standing partnership? Am J Physiol Endocrinol Metab 298: E751–760. doi: 10.1152/ajpendo.00745.2009 PMID: 20103737

22. Kim D, Nguyen MD, Dobbin MM, Fischer A, Sananbenesi F, Rodgers JT, et al. (2007) SIRT1 deacetylase protects against neurodegeneration in models for Alzheimer's disease and amyotrophic lateral sclerosis. EMBO J 26: 3169–3179. PMID: 17581637

23. Donmez G, Arun A, Chung CY, McLean PJ, Lindquist S, Guarente L (2012) SIRT1 protects against alpha-synuclein aggregation by activating molecular chaperones. J Neurosci 32: 124–132. doi: 10.1523/JNEUROSCI.3442-11.2012 PMID: 22219275

24. Parker JA, Arango M, Abderrahmane S, Lambert E, Tourette C, Catoire H, et al. (2005) Resveratrol rescues mutant polyglutamine cytotoxicity in nematode and mammalian neurons. Nat Genet 37: 349–350. PMID: 15793589

25. Jiang M, Wang J, Fu J, Du L, Jeong H, West T, et al. (2012) Neuroprotective role of Sirt1 in mammalian models of Huntington's disease through activation of multiple Sirt1 targets. Nat Med 18: 153–158.

26. Jeong H, Cohen DE, Cui L, Supinski A, Savas JN, Mazzulli JR, et al. (2012) Sirt1 mediates neuroprotection from mutant huntingtin by activation of the TORC1 and CREB transcriptional pathway. Nat Med 18: 159–165.

27. Jiang M, Zheng J, Peng Q, Hou Z, Zhang J, Mori S, et al. (2014) Sirtuin 1 activator SRT2104 protects Huntington's disease mice. Ann Clin Transl Neurol 1: 1047–1052. doi: 10.1002/acn3.135 PMID: 25574479

28. Hoffmann E, Wald J, Lavu S, Roberts J, Beaumont C, Haddad J, et al. (2013) Pharmacokinetics and tolerability of SRT2104, a first-in-class small molecule activator of SIRT1, after single and repeated oral administration in man. Br J Clin Pharmacol 75: 186–196. doi: 10.1111/j.1365-2125.2012.04340.x PMID: 22616762

29. Smith MR, Syed A, Lukacsovich T, Purcell J, Barbaro BA, Worthge SA, et al. (2014) A potent and selective Sirtuin 1 inhibitor alleviates pathology in multiple animal and cell models of Huntington's disease. Hum Mol Genet 23: 2995–3007. doi: 10.1093/hmg/ddu010 PMID: 24436303

30. Sussmuth SD, Haider S, Landwehrmeyer GB, Farmer R, Frost C, Tripepi G, et al. (2015) An exploratory double-blind, randomized clinical trial with selisistat, a SirT1 inhibitor, in patients with Huntington's disease. Br J Clin Pharmacol 79: 465–476. doi: 10.1111/bcp.12512 PMID: 25223731

31. Kim JE, Chen J, Lou Z (2008) DBC1 is a negative regulator of SIRT1. Nature 451: 583–586. doi: 10.1038/nature06500 PMID: 18235501

32. Sathasivam K, Lane A, Legleiter J, Warley A, Woodman B, Finkbeiner S, et al. (2010) Identical oligomeric and fibrillar structures captured from the brains of R6/2 and knock-in mouse models of Huntington's disease. Hum Mol Genet 19: 65–78. doi: 10.1093/hmg/ddp467 PMID: 19825844

33. Li H, Rajendran GK, Liu N, Ware C, Rubin BP, Gu Y (2007) SirT1 modulates the estrogen-insulin-like growth factor-1 signaling for postnatal development of mammary gland in mice. Breast Cancer Res 9: R1. PMID: 17201918

34. Satoh A, Brace CS, Ben-Josef G, West T, Wozniak DF, Holtzman DM, et al. (2010) SIRT1 promotes the central adaptive response to diet restriction through activation of the dorsomedial and lateral nuclei of the hypothalamus. J Neurosci 30: 10220–10232. doi: 10.1523/JNEUROSCI.1385-10.2010 PMID: 20668205

35. Hockly E, Richon VM, Woodman B, Smith DL, Zhou X, Rosa E, et al. (2003) Suberoylanilide hydroxamic acid, a histone deacetylase inhibitor, ameliorates motor deficits in a mouse model of Huntington's disease. Proc Natl Acad Sci U S A 100: 2041–2046. PMID: 12576549

36. Hockly E, Tse J, Barker AL, Moolman DL, Beunard JL, Revington AP, et al. (2006) Evaluation of the benzothiazole aggregation inhibitors riluzole and PGL-135 as therapeutics for Huntington's disease. Neurobiol Dis 21: 228–236. PMID: 16111888

37. Hockly E, Woodman B, Mahal A, Lewis CM, Bates G (2003) Standardization and statistical approaches to therapeutic trials in the R6/2 mouse. Brain Res Bull 61: 469–479. PMID: 13679245

38. Benn CL, Fox H, Bates GP (2008) Optimisation of region-specific reference gene selection and relative gene expression analysis methods for pre-clinical trials of Huntington's disease. Mol Neurodegener 3: 17. doi: 10.1186/1750-1326-3-17 PMID: 18954449

39. Langley E, Pearson M, Faretta M, Bauer UM, Frye RA, Minucci S, et al. (2002) Human SIR2 deacetylates p53 and antagonizes PML/p53-induced cellular senescence. EMBO J 21: 2383–2396. PMID: 12006491

40. Solomon JM, Pasupuleti R, Xu L, McDonagh T, Curtis R, DiStefano PS, et al. (2006) Inhibition of SIRT1 catalytic activity increases p53 acetylation but does not alter cell survival following DNA damage. Mol Cell Biol 26: 28–38. PMID: 16354677

41. Sasaki T, Maier B, Koclega KD, Chruszcz M, Gluba W, Stukenberg PT, et al. (2008) Phosphorylation regulates SIRT1 function. PLoS One 3: e4020. doi: 10.1371/journal.pone.0004020 PMID: 19107194

42. Westendorf JM, Rao PN, Gerace L (1994) Cloning of cDNAs for M-phase phosphoproteins recognized by the MPM2 monoclonal antibody and determination of the phosphorylated epitope. Proc Natl Acad Sci U S A 91: 714–718. PMID: 8290587

43. Johnson LN (2009) The regulation of protein phosphorylation. Biochem Soc Trans 37: 627–641. doi: 10.1042/BST0370627 PMID: 19614568

44. Nasrin N, Kaushik VK, Fortier E, Wall D, Pearson KJ, de Cabo R, et al. (2009) JNK1 phosphorylates SIRT1 and promotes its enzymatic activity. PLoS One 4: e8414. doi: 10.1371/journal.pone.0008414 PMID: 20027304

45. Lau AW, Liu P, Inuzuka H, Gao D (2014) SIRT1 phosphorylation by AMP-activated protein kinase regulates p53 acetylation. Am J Cancer Res 4: 245–255. PMID: 24959379

46. Muchowski PJ, Wacker JL (2005) Modulation of neurodegeneration by molecular chaperones. Nat Rev Neurosci 6: 11–22. PMID: 15611723

47. Morley JF, Morimoto RI (2004) Regulation of longevity in Caenorhabditis elegans by heat shock factor and molecular chaperones. Mol Biol Cell 15: 657–664. PMID: 14668486

48. Douglas PM, Summers DW, Cyr DM (2009) Molecular chaperones antagonize proteotoxicity by differentially modulating protein aggregation pathways. Prion 3: 51–58. PMID: 19421006

49. Steffan JS, Bodai L, Pallos J, Poelman M, McCampbell A, Apostol BL, et al. (2001) Histone deacetylase inhibitors arrest polyglutamine-dependent neurodegeneration in Drosophila. Nature 413: 739–743. PMID: 11607033

50. Zschoernig B, Mahlknecht U (2009) Carboxy-terminal phosphorylation of SIRT1 by protein kinase CK2. Biochem Biophys Res Commun 381: 372–377. doi: 10.1016/j.bbrc.2009.02.085 PMID: 19236849

51. Bae BI, Xu H, Igarashi S, Fujimuro M, Agrawal N, Taya Y, et al. (2005) p53 mediates cellular dysfunction and behavioral abnormalities in Huntington's disease. Neuron 47: 29–41. PMID: 15996546
